# The ethnic density effect in psychosis: a systematic review and multilevel meta-analysis

**DOI:** 10.1192/bjp.2021.96

**Published:** 2021-12

**Authors:** Sophie J. Baker, Mike Jackson, Hannah Jongsma, Christopher W. N. Saville

**Affiliations:** School of Psychology, Bangor University, UK; North Wales Clinical Psychology Programme, School of Psychology, Bangor University; and Betsi Cadwaladr University Health Board, Bangor, UK; Centre for Transcultural Psychiatry Veldzicht, Balkbrug; and Department of Psychiatry, University of Groningen; and University Medical Centre Groningen, The Netherlands; North Wales Clinical Psychology Programme, School of Psychology, Bangor University, UK

**Keywords:** Ethnic density, psychosis, schizophrenia, minority groups, mental health inequality

## Abstract

**Background:**

An ‘ethnic’ or ‘group’ density effect in psychosis has been observed, whereby the risk of psychosis in minority group individuals is inversely related to neighbourhood-level proportions of others belonging to the same group. However, there is conflicting evidence over whether this effect differs between minority groups and limited investigation into other moderators.

**Aims:**

To conduct a comprehensive systematic review and meta-analysis of the group density effect in psychosis and examine moderators.

**Method:**

Four databases were systematically searched. A narrative review was conducted and a three-level meta-analysis was performed. The potential moderating effect of crudely and specifically defined minority groups was assessed. Country, time, area size and whether studies used clinical or non-clinical outcomes were also tested as moderators.

**Results:**

Thirty-two studies were included in the narrative review and ten in the meta-analysis. A 10 percentage-point decrease in own-group density was associated with a 20% increase in psychosis risk (OR = 1.20, 95% CI 1.09−1.32, *P* < 0.001). This was moderated by crudely defined minority groups (*F*_6,68_ = 6.86, *P* < 0.001), with the strongest associations observed in Black populations, followed by a White Other sample. Greater heterogeneity was observed when specific minority groups were assessed (*F*_25,49_ = 7.26, *P* < 0.001).

**Conclusions:**

This is the first review to provide meta-analytic evidence that the risk of psychosis posed by lower own-group density varies across minority groups, with the strongest associations observed in Black individuals. Heterogeneity in effect sizes may reflect distinctive social experiences of specific minority groups. Potential mechanisms are discussed, along with the implications of findings and suggestions for future research.

Compared with their majority counterparts, ethnic minority and migrant groups are at greater risk of mental health difficulties,^1^ particularly psychosis.^2–5^ This excess risk is not observed in migrant groups’ countries of origin,^6^ nor can it be explained by diagnostic biases or genetic risk factors.^7^ Interestingly, this elevated risk is context-dependent, such that minority group members living in neighbourhoods with a low proportion of their group are more likely to experience psychosis than those residing in areas where their group is well represented.^8^ This association, termed the ‘ethnic’ or ‘group’ density effect, operates in a dose–response manner,^9^ holds after adjustment for socioeconomic deprivation^10^ and is proposed to act as a buffer against social disadvantages experienced by minorities.^11^ Most studies have focused on minorities classified by their ethnicity or migratory background, but poorer mental health has also been observed in minority groups defined by other characteristics, including sexuality,^12,13^ political affiliation^14^ and religion.^15^ As the present review will include minorities grouped by other ‘non-ethnic’ social characteristics in addition to ethnic minorities and migrants, hereafter we will use the term ‘group density’ instead of ‘ethnic density’.

So far, there have been three reviews of the group density effect in psychosis,^10,16,17^ all of which examined associations in ethnic minorities and migrants. At present, it is unclear whether lower own-group density areas confer the same risk across different minority groups. The most recent meta-analysis found that ethnic group did not moderate group density associations.^16^ However, narrative reviews noted that studies examining pooled ethnic minority samples tended to report more consistent effects than studies assessing specific minorities, which yielded mixed results.^10,17^ Specific marginalised and minority groups have distinct social experiences, so investigating group density relationships in combined samples might mask important group differences.^10,11^ Identifying heterogeneity in effect sizes between different minority groups, ethnic and otherwise, may elucidate potential causal mechanisms.^18^ More broadly, identifying moderators of this phenomenon is important for understanding the aetiological underpinnings of psychotic disorders and for providing targeted clinical and policy interventions for minorities.^7,10,16^ In this review, we aim to conduct a comprehensive systematic review and meta-analysis of the group density effect in psychosis and examine potential moderators, particularly those associated with specific minority groups.

## Method

We followed Preferred Reporting Items for Systematic Reviews and Meta-Analyses (PRISMA)^19^ and Meta-analysis of Observational Studies in Epidemiology (MOOSE) guidelines.^20^ The protocol for this review was pre-registered on PROSPERO (reference: CRD42019139384). Deviations from protocol can be found in Supplemental 1 of the Supplementary material available at https://doi.org/10.1192/bjp.2021.96.

### Search strategy

In May 2019, S.J.B. conducted electronic searches of four databases (PsycINFO, Web of Science, PubMed and CINAHL Plus). Searches were repeated in August 2020. We consulted with Bangor University's academic support librarian for the College of Human Sciences for assistance with designing the search strategies. The search strategies were piloted before the final search was executed. Each search utilised truncation and thesaurus tools to find related terms and enhance retrieval of relevant articles. The full list of search terms and an example search strategy for one database can be found in Supplementals 2 and 3. Below is an example of the organisation of search terms:

- population, e.g. Psychosis OR Psychotic OR Schizophrenia OR Bipolar
- ethnic density-related terms, e.g. ‘Ethnic density’ OR ‘Group density’ OR ‘Ethnic composition’ OR ‘Ethnic enclave’
- outcome measures, e.g. Incidence OR Prevalence OR Symptom* OR ‘Ultra-high risk’
- geographical terms, e.g. Neighbo* OR Municipal OR ‘Electoral ward’ OR ‘Output area’

A AND B AND C AND D.

### Eligibility criteria

For the narrative review, we included any peer-reviewed primary study examining the group density effect in psychosis. For the meta-analysis, additional criteria were applied, as follows:

- primary epidemiological studies assessing a within-group density association, i.e. comparing psychosis risk within minority groups between different levels of group density
- geographical units averaged 50 000 people or fewer
- group density exposure quantified using census data or similar
- validated quantitative instrument(s) used to measure psychosis outcomes, including incident cases, psychosis experiences, prodromal psychosis or symptomatology
- studies reported odds ratios (ORs), incidence rate ratios (IRRs), hazard ratios (HRs) or relative risks (RRs), effect size measures and 95% confidence intervals (CIs)
- studies used multilevel modelling to account for non-independence of data
- studies adjusted for individual- and area-level confounds (minimally, age, gender and area-level deprivation).

### Study selection and data extraction

Articles were exported to Mendeley citation management software. After removing duplicates, S.J.B. and C.W.N.S. independently assessed all titles and abstracts for eligibility and any papers that either author deemed relevant were carried forward to the next stage of screening. Kappa indicated substantial agreement between authors (*k* = 0.754).

Full texts of remaining articles were independently screened by S.J.B. and C.W.N.S., with 100% agreement regarding which studies should be included in the narrative review and meta-analysis components of the review. Uncertainties concerning eligibility were resolved through discussion or contacting authors where clarification was needed. Reference sections were hand-searched to identify any further papers. For potentially relevant articles that were not available in English, we assessed eligibility by translating the article or contacting the first author of the paper (Supplemental 4). Study characteristics and meta-data from included studies were extracted. Authors were contacted for additional data where necessary. For studies with overlapping data-sets, only the study with the largest sample was included in the meta-analysis. If samples were equally large, we included the study where group categories were most compatible with other studies.

### Data analysis

A narrative review was conducted and studies meeting inclusion criteria were included in the meta-analysis. As group density studies often include dependent effect sizes – multiple samples (level 2) nested within studies (level 3) – we used ‘multilevel’ meta-analysis,^21–23^ as implemented using the rma.mv function in the R package Metafor^24,25^ to appropriately control error rates.^23^

Effect sizes with CIs were extracted from the fully adjusted models in each paper. As studies quantified exposure differently, we rescaled effect sizes to reflect decreases of 10 percentage points in group density. Effect sizes and CIs were then converted to their natural logarithmic form, from which log standard errors and sampling variances were computed. See Supplemental 5 for further information on how effect sizes were rescaled.

The three-level model was fitted to estimate the overall pooled effect size. To assess fit, we reran the analysis twice, holding the variance component of level 2 or level 3 constant.^23^ Akaike information criteria for full and reduced models were compared to assess fit.

The overall pooled effect size comprised all samples. Separate pooled effect sizes were computed for groups defined by ethnicity or migratory background, minority groups classified by other characteristics, and neighbourhood studies only.

We additionally examined *a priori* hypothesised moderators and the effect of removing individual studies and samples on the pooled effect. For each moderator test, the most common grouping was used as the reference category. To derive subgroups, the 18-group self-ascribed classification system for ethnic groups used by the 2011 UK Census was used to allocate samples into ‘crude minority groups’ (the UK was the most common study setting). Subgroups for the ‘specific minority groups’ moderator test were informed by the most specific minority group categories reported by the authors of the studies. To assess the moderating effect of area sizes, we calculated area size quartiles using reported average area sizes. If average area sizes were not available, census data were used to derive an estimate. We also stratified data by the geographic unit used: lower super output area (LSOA) or smaller and all other area sizes.

We used a quality assessment tool developed for ethnic density studies specifically, which has been used in a previous review^16^ (Supplemental 6). We additionally conducted GRADE assessments to evaluate the evidence for each psychosis outcome and crude minority subgroup (Supplemental 7).

## Results

**Fig. 1:**
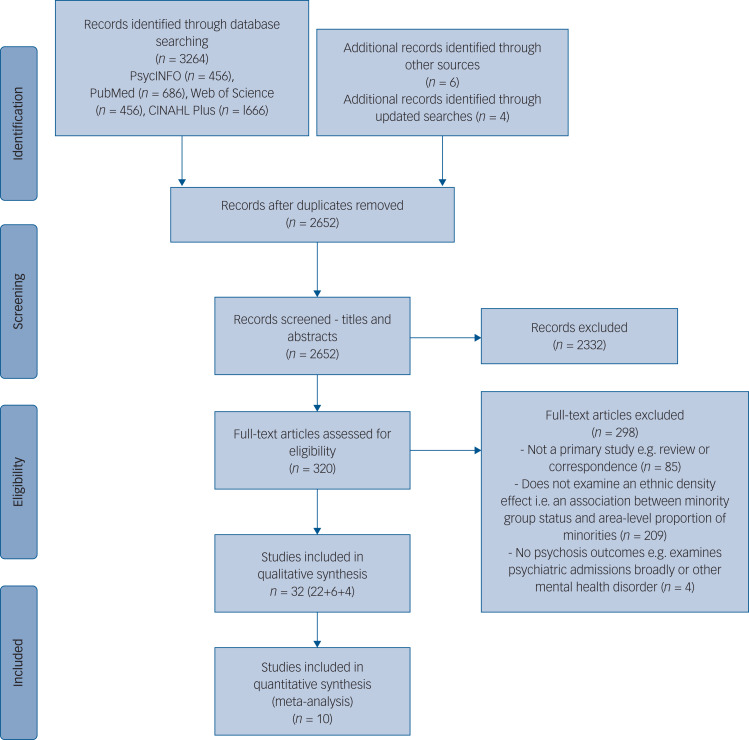
PRISMA diagram outlining study selection procedure.

**Fig. 2:**
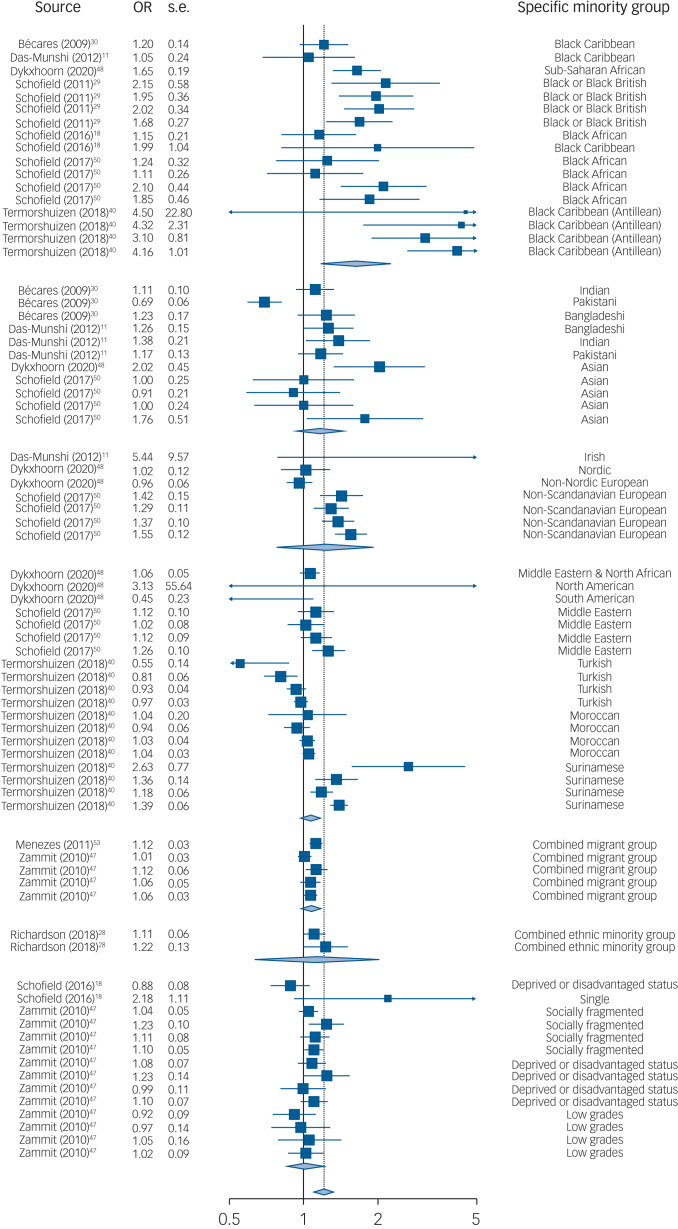
Forest plot of the association between a 10 percentage-point decrease in group density and psychosis risk

The search identified 2652 unique articles, and 32 studies were included in the narrative review (Fig. 1). Ten studies met inclusion criteria for the meta-analysis, comprising 75 samples. Each sample contributed <2% weighting to the overall pooled effect size (Fig. 2 shows the forest plot).

### Narrative review

#### Study characteristics

Fourteen studies (44%) were conducted in the UK,^9,11,18,26–36^ nine (28%) in The Netherlands^37–45^ and four (13%) in Sweden.^46–49^ Of the remaining five (16%), two were conducted in Denmark^50,51^ and one each in the USA,^52^ Canada^53^ and Australia.^54^

The majority were retrospective epidemiological studies (*n* = 26, 81%).^9,11,18,26–36,39–42,46–51,53,54^ Of these, most were cross-sectional but six (four data-sets) were longitudinal.^33,47–51^ All these studies were conducted in a neighbourhood context except one, which used a school setting.^47^ The other six studies examined virtual reality (VR) environments,^43,44^ *perceived* ethnic density,^52^ symptomatology,^37,38^ remission,^38^ and ‘bully climate’.^45^

Around half of the studies (*n* = 15, 47%) examined first incident cases^9,26–29,32,36,39,41,42,46–48,50,51^ and seven (22%) used measures of subclinical psychosis.^11,18,30,31,34,45,52^ The others assessed symptomatic outcomes,^37,38,43,44^ mortality rates,^33^ length of admission,^35^ compulsory admission,^49^ individuals meeting ultra-high risk (UHR) criteria,^54^ dispensed antipsychotic medication,^40^ lifetime prevalence of psychosis,^53^ psychophysiological outcomes^43^ and interpersonal distance.^43,44^ Study characteristics are summarised in Supplementary Table 1.

### Summary of results by minority group sample

#### Combined ethnic minority or migrant groups

Seventeen studies (fifteen data-sets) reported associations for aggregated minority ethnic or migrant groups.^9,11,26,28,30,33–36,39,42,46–49,53,54^ In combined minority groups in the UK^9,11,26,28,30,34,36^ and migrant groups in Sweden,^46–48^ The Netherlands^39,42^ and Canada,^53^ all but one study^46^ found associations in the expected direction for clinical^9,26,28,36,39,42,47,48,53^ and non-clinical outcomes,^11,30,34^ with many finding significant relationships.^9,11,34,39,42,47,48^ (Another study in The Netherlands^55^ examined the relationship between group density and perceived discrimination in a migrant sample of individuals with psychosis and controls.) Between-group density effects tended to be stronger than within-group effects^36,39^ and one study found a significant association for affective but not non-affective psychosis.^28^ For other outcomes, significant associations were observed for mortality rates^33^ and compulsory admission,^49^ but not for duration of admission^35^ or meeting UHR criteria.^54^

#### Black populations

Fourteen studies (twelve data-sets) included Black individuals.^11,18,27,29–34,39,40,48,50,51^ Significant group density associations were found in aggregated Black clinical and non-clinical samples in the UK.^18,29^ In Black Caribbean populations,^11,18,27,30–34^ significant results were observed for subclinical psychosis^18,34^ and schizophrenia first incident cases^32^ in the UK, and strong associations were consistently observed in Antillean individuals for non-affective psychosis^39^ and prescribed antipsychotics^40^ in The Netherlands. Other UK studies reported weaker or no evidence of associations in Caribbean groups for subclinical psychosis,^11,30,31^ non-affective psychosis^27^ and mortality rates.^33^ In Black African individuals,^18,27,32,33,49–51^ a strong association between ethnic density during adolescence and later psychosis was observed in Denmark^50,51^ and Sweden,^48^ with one study finding stronger associations in second-generation^51^ and the other in first-generation African migrants.^48^ In the UK, a significant relationship was found for Black African individuals and non-affective psychosis.^27^ Other UK studies found no significant associations in Black African groups,^18,32,33^ although one found weak evidence of an association for all-cause mortality (*P* = 0.068).^33^

#### Asian populations

Eight studies (seven data-sets) examined Asian populations.^11,27,30,31,33,34,48,50^ In combined Asian groups, consistent associations between own-group density and non-affective psychosis were observed in Denmark^50^ and Sweden,^48^ with the latter demonstrating a stronger relationship in first-generation Asian migrants.^48^ There was also a strong association with all-cause mortality rates in the UK.^33^ When considering Asian subgroups, UK studies^11,27,30,31,34^ found associations in the expected direction in Indian and Bangladeshi groups for subclinical psychosis,^11,30,31,34^ although one study examining first incident psychosis cases reported no evidence of a relationship in Bangladeshi individuals.^27^ Only one study included African Asian and Chinese samples,^34^ no significant correlations were found for either group. In Pakistani individuals, no study found evidence of an association,^11,30,31,34^ with two studies noting detrimental relationships.^30,34^

#### White Other populations

Seven studies (five data-sets) reported results for White Other samples.^11,27,31,33,48,50,51^ In the UK, associations were in the expected direction but non-significant in Irish individuals for subclinical psychosis,^11,31^ and no evidence for a relationship was observed in Irish individuals for mortality rates^33^ or in a non-British White sample for non-affective psychosis.^27^ There was also no association in non-Swedish Nordic or non-Nordic European migrants in Sweden.^48^ However, in Denmark, significant relationships were found in non-Scandinavian European groups for non-affective psychosis,^50^ with negligible differences between first- and second-generation migrants.^51^

#### Other ethnic groups

Seven studies (six data-sets) included other ethnic minority and migrant groups.^39,40,42,46,48,50,51^ Longitudinal analyses in Denmark found significant relationships in Middle Eastern individuals for non-affective psychosis,^50^ with stronger associations for second-generation migrants.^51^ However, in a Middle Eastern and North African sample in Sweden, there was no significant relationship between own-group density at age 15 and later risk of psychosis.^48^ The same study found no associations in North American, South American, Swedish and Mixed migrants, with some groups in fact showing (non-significant) detrimental relationships.^48^ Another Swedish study found no difference in non-affective psychosis risk between Iraqi migrants living in ethnic enclaves and those in predominantly Swedish areas.^46^ In migrant groups in The Netherlands, associations were consistently strong for a combined Surinamese/Antillean group and a Surinamese only sample for both non-affective psychosis^39^ and antipsychotic usage^40^ respectively. However, results were mixed for Turkish and Moroccan groups.^39,40,42^

#### Other social characteristics

Three studies included minority groups classified by characteristics other than ethnicity or migratory background,^18,41,47^ namely single marital/household status,^18,41^ disadvantaged social class,^18,47^ social fragmentation^47^ and low academic grades.^47^ Significantly increased risk of schizophrenia was observed in single individuals living in neighbourhoods with fewer single people in The Netherlands.^41^ This was also observed in individuals in single households in a later UK study, but the relationship was non-significant.^18^ A longitudinal study in Sweden assessing associations between school-level own-group density and clinical psychosis found a significant association in socially fragmented groups, but not in those with low grades or deprived status, although the latter approached significance (*P* = 0.057).^47^ A relationship for disadvantaged status was not found in the UK neighbourhood-level study, which showed a (non-significant) reverse association.^18^

#### Virtual reality, symptomatology, perceived ethnic density and bully climate

Six studies used different methods: two used VR,^43,44^ two looked at symptom profiles^37,38^ and remission,^38^ one examined perceived ethnic density^52^ and one considered ‘bully climate’.^45^ VR studies simulated high and low group density environments by manipulating the ethnicity of avatars.^43,44^ Compared with control participants, individuals with psychosis had higher galvanic skin responses in low own-group density conditions.^43^ The second study found no effect of virtual group density on distress or paranoid thoughts.^44^ (Veling and colleagues used the VR experiment^44^ also to examine the effect of virtual social stressors (including minority status) on individuals with differing psychosis liability using additional outcomes such as autonomic balance,^56^ Th17/T regulator cell balance and natural killer cell numbers^57^ and interpersonal distance.^58^ Moderators including cognitive biases^59^, self-esteem^60^ and childhood trauma^61^ have also been investigated.)

In symptom studies, an ethnic density interaction for paranoia was observed in ethnic majority, but not ethnic minority, adolescents in a Dutch classroom setting,^37^ whereas another study found no association between group density and symptomatic outcomes.^38^

The perceived ethnic density study found that Black, Latino and Asian individuals in the USA who reported growing up in neighbourhoods with higher proportions of out-group ethnic minority individuals reported more psychotic-like symptoms than those who grew up in ethnically concordant or predominantly White neighbourhoods.^52^ Further, Black individuals who perceived a change in the ethnic density of their neighbourhood during childhood reported more psychotic experiences than those who did not.^52^

The remaining study examined a group density association for bullying in a classroom setting. Individuals who both bullied others and were victims of bullying reported the highest subclinical psychotic experiences compared with bullies, victims and children not involved in bullying. The association between bully-victim status and psychosis was attenuated by a higher ‘bully climate’, i.e. classrooms with higher proportions of other children involved in bullying in some capacity.^45^

### Meta-analysis

Ten studies were eligible for meta-analysis.^11,18,28–30,40,47,48,50,53^ Of the twenty-two studies excluded, six studies^26,31,32,36,39,51^ used overlapping or potentially overlapping data-sets,^11,29,40,50^ five used non-eligible outcomes,^33,35,37,38,49^ four used non-eligible exposures (VR simulation,^43,44^ perceived ethnic density^52^ and ethnic enclaves^46^). Four did not adjust for the specified individual and area-level confounds,^27,41,45,54^ two only examined between-group density effects^9,42^ and one did not use multilevel modelling.^34^

Although Schofield and colleagues^51^ were the first to examine generational differences in the group density effect, their study used the same cohort as another study^50^ and, as per the eligibility criteria, we included their earlier study as it included an additional minority group sample (Asian).^50^ This meant that Dykxhoorn et al^48^ was the only included paper that stratified results by generational status, so only data for first-generation migrants were extracted from this study.

#### Pooled group density effects

The three-level model was the best fit for the data (Supplemental 8). The overall meta-analytic effect indicated that a 10 percentage-point decrease in group density was associated with a 20% increase in psychosis risk (OR = 1.20, 95% CI 1.09−1.32, *P* < 0.001). An estimate using only minority groups defined by ethnicity or migratory background was also significant (OR = 1.23, 95% CI 1.14−1.33, *P* < 0.001). There was no significant effect in minority groups defined by other characteristics (OR = 1.02, 95% CI 0.86−1.20, *P* = 0.848). Results were similar after removal of the one school-based study^47^ (OR = 1.25, 95% CI 1.15−1.36, *P* < 0.001).

#### Moderator tests

In line with the narrative review, there were moderating effects of crude (*F*_6,68_ = 6.86, *P* < 0.001) and specific minority groups (*F*_25,49_ = 7.26, *P* < 0.001). Said moderator tests were also significant when conducted on ethnic minority and migrant samples only (Supplemental 9). Further analyses examining whether associations differed when minority groups were self-ascribed or defined by birthplace were non-significant (*F*_1,59_ = 0.60, *P* = 0.443).

When assessing crude minority groups, the strongest association was observed in the Black group (OR = 1.71, 95% CI 1.43−2.03, *P* < 0.001) relative to the reference group (‘Other ethnic group’). There was also a stronger association in the White Other group (OR = 1.23, 95% CI 1.03−1.48, *P* = 0.024). There was weak evidence of a stronger association in Asian populations (OR = 1.19, 95% CI 0.98−1.45, *P* = 0.074).

Moderator tests for specific minority groups showed the strongest associations in Black Antillean migrants in The Netherlands (OR = 3.60, 95% CI 2.22−5.83, *P* < 0.001) relative to the reference group (‘Combined migrant group’). This was followed by Black or Black British (OR = 1.84, 95% CI 1.24−2.74, *P* = 0.003) and Black African (OR = 1.48, 95% CI 1.10−2.00, *P* = 0.011) groups in the UK and Denmark. There was also a stronger association in the non-Scandinavian European group (OR = 1.43, 95% CI 1.06−1.92, *P* = 0.020) and a significant reversed association in a South American sample (OR = 0.37, 95% CI 0.14−0.99, *P* = 0.048). Table 1 shows moderator test results including crude minority groups, and Supplemental 10 shows specific minority group results.

**Table 1:** Moderator tests

Variable	Samples, *n*	Studies, *n*	Pooled OR (95% CI)	s.e.	*P*
Country	−	–	*F*_4,70_ = 0.18	−	0.946
Sweden	23	2	1	−	−
UK	19	5	1.13 (0.81−1.58)	0.17	0.456
Canada	1	1	1.01 (0.56−1.82)	0.30	0.983
Denmark	16	1	1.15 (0.73−1.81)	0.23	0.550
The Netherlands	16	1	1.07 (0.68−1.68)	0.23	0.774
Median time cases collected	−	−	*F*_2,72_ = 1.25	−	0.292
1990s and earlier	43	4	1	−	−
2010s	18	2	1.04 (0.82−1.32)	0.12	0.752
2000s	14	4	1.19 (0.95−1.49)	0.11	0.121
Area size	−	−	*F*_3,71_ = 2.50	−	0.066
1st quartile (<920)	24	3	1	−	−
2nd quartile (920–2532)	8	2	1.38 (1.08−1.77)	0.12	0.011*
3rd quartile (2532–4993)	18	2	1.14 (0.95−1.37)	0.09	0.168
4th quartile (4993–7200)	25	3	1.06 (0.89−1.25)	0.84	0.513
LSOA or larger	−	−	*F*_1,73_ = 0.13	−	0.723
≤LSOA	32	5	1	−	−
>LSOA	43	5	0.96 (0.78−1.19)	0.11	0.723
Psychosis outcome	−	−	*F*_5,69_ = 2.36	−	0.049*
Non-affective psychosis	33	5	1	−	−
Subclinical psychosis experiences	13	3	0.97 (0.80−1.17)	0.10	0.753
Antipsychotic prescriptions	16	1	1.02 (0.84−1.25)	0.10	0.817
Any psychosis	4	1	1.66 (1.22−2.27)	0.16	0.002*
Affective psychosis	5	2	1.04 (0.84−1.30)	0.11	0.695
Other psychoses	4	1	0.97 (0.78−1.22)	0.11	0.818
Clinical or non-clinical outcome	−	−	*F*_1,73_ = 0.59	−	0.444
Clinical	62	7	1	−-	−
Non-clinical	13	3	0.92 (0.73−1.15)	0.12	0.444
Crude minority groups	−	−	F_6,68_ = 6.86	−	<0.001*
Other ethnic group	19	3	1	−	−
Asian	11	4	1.19 (0.98−1.45)	0.10	0.074
Black	17	7	1.71 (1.43−2.03)	0.09	<0.001*
Combined ethnic minority group	2	1	1.17 (0.83−1.65)	0.17	0.355
Combined migrant group	5	2	1.09 (0.86−1.39)	0.12	0.476
Other social characteristic	14	2	1.09 (0.87−1.37)	0.12	0.463
White Other	7	3	1.23 (1.03−1.48)	0.09	0.024*
Minority group allocation	–	–	*F*_1,59_ = 0.60	−	0.443
Birthplace of individual or parents	44	5	1	−	−
Self-ascribed ethnic minority	17	5	1.08 (0.88−1.33)	0.10	0.443

LSOA, lower super output area.

* *P < 0.05*.

Moderator tests for country, time and area size were non-significant, although there was some evidence for stronger group density associations at smaller geographic units. There was also a significant moderating effect of psychosis outcome used (*F*_5,69_ = 2.36, *P* = 0.049), with evidence for stronger associations in studies using clinical outcomes, namely non-affective psychosis cases (OR = 1.15, 95% CI 1.04−1.28, *P* = 0.008) and cases with a first diagnosis of any psychotic disorder (OR = 1.66, 95% CI 1.22−2.27, *P* = 0.002).

#### Sensitivity analyses

Leave-one-out analysis indicated that removing each study produced negligible changes to the overall pooled effect (Table 2). This was also the case when the 75 effect sizes within the studies were individually removed (Supplemental 11).

**Table 2:** Effect sizes by study and leave-one-out analysis

Study first author and year, setting	Minority groupings: crude group (specific group, cases/total)	Study quality	Pooled OR (95% CI)	Pooled OR (95% CI) if study removed	*P* if study removed
All studies (*n* = 10)	–	–	1.20 (1.09−1.32), *P* < 0.001	–	–
Bécares (2009)^30^, UK	Black (Black Caribbean: n.r./1215)	14	1.04 (0.84−1.27)	1.22 (1.10−1.35)	<0.001
	Asian (Indian: n.r./1278; Pakistani: n.r./1190; Bangladeshi: n.r./594)				
Das-Munshi (2012)^11^, UK	Black (Black Caribbean: 83/694)	15	1.63 (0.87−3.05)	1.20 (1.08−1.33)	0.001
	Asian (Indian 58/643; Pakistani 72/724; Bangladeshi 33/650)				
	White Other (Irish: 59/733)				
Dykxhoorn (2020)^48^, Sweden	Black (sub-Saharan African: 550/261 899 person-years)	14	1.25 (0.51−3.06)	1.20 (1.08−1.34)	0.001
	Asian (Asian: 297/365 971)				
	Other ethnic group (Middle Eastern & North African: 693/796 928; North American: 50/55 558; South American: 79/102 857)				
	White Other (Nordic: 103/131 882; non-Nordic European: 693/880 211)				
Menezes (2011)^53^, Canada	Combined migrant group (combined migrant group: 31/7784)	11	1.12 (1.05−1.19)	1.20 (1.09−1.33)	<0.001
Richardson (2018)^28^, UK	Combined ethnic minority group (combined ethnic minority group: 160/398 511 person-years)	12	1.16 (1.00−1.36)	1.20 (1.09−1.34)	<0.001
Schofield (2011)^29^, UK	Black (Black or Black British: 109/23 693)	11	1.94 (1.34−2.82)	1.15 (1.08−1.23)	<0.001
Schofield (2016)^18^, UK	Black (Black African: n.r./234; Black Caribbean: n.r./143; overall: 98/377)	11	1.45 (0.81−2.57)	1.21 (1.09−1.34)	<0.001
	Other social characteristic (deprived or disadvantaged: 101/421; single marital status: 51/212)				
Schofield (2017)^50^, Denmark	Black (African: 362/13 118)	12	1.28 (0.94−1.75)	1.19 (1.07−1.33)	0.002
	Asian (Asian: 415/24 512)				
	Other ethnic group (Middle Eastern: 529/28 762)				
	White Other (non-Scandinavian European: 1175/58 939)				
Termorshuizen (2018)^40^, The Netherlands	Black (Antillean: 949/41 430)	11	1.49 (0.96−2.34)	1.19 (1.06−1.33)	0.005
	Other ethnic group (Turkish: 3775/105 460; Moroccan: 5207/115 455; Surinamese: 4252/147 123)				
Zammit (2010)^47^, Sweden	Combined migrant group (combined migrant group, n.r.)	13	1.07 (0.92−1.24)	1.25 (1.15−1.36)	<0.001
	Other social characteristic (deprived or disadvantaged: n.r.; socially fragmented: n.r.; low grades: n.r.)				

n.r., not recorded.

## Discussion

### Summary

This is the first review providing quantitative evidence that the risk of psychosis posed by lower own-group density areas varies across minority groups. Overall, a 10 percentage-point decrease in own-group density was associated with a 20% increase in risk of psychosis, but this effect was strongly moderated by minority group.

### Comparisons with previous reviews

Our overall pooled effect size estimate was similar in magnitude to a previous within-groups meta-analysis^16^ but weaker than one examining between-group effects.^10^ However, contrary to previous analyses,^16^ we observed a strong moderating effect of minority group, particularly when more fine-grained classifications were tested.

In line with previous narrative reviews,^10,17^ we observed the strongest group density associations in Black individuals. A significantly stronger association was also found in the White Other group, driven by strong associations in non-Scandinavian European individuals in Denmark.^50^ A reverse relationship was noted in South American migrants to Sweden.^48^ Such heterogeneity in effect sizes may reflect distinctive social experiences of specific minority groups.^8,11^

### Strengths and limitations

A strength of this review is our use of a multilevel meta-analytic model. In the group density literature, it is common to examine multiple groups and so accounting for nesting by study is vital. Another strength is that we used relatively specific minority groupings. It is common practice for group density studies to amalgamate minority samples (e.g. Black and minority ethnic groups), for reasons of statistical power.^18^ As we show, aggregating groups may conceal considerable heterogeneity in risk. This likely reflects distinct social experiences of different minority groups, in turn providing clues to likely mechanisms. For example, the narrative review and meta-analysis indicate that reduced ethnic density confers greater risk to Black populations compared with other groups. However, within the Black group, associations appear stronger in Black Caribbean individuals in The Netherlands than in the UK, highlighting the importance of the varied experiences of different migrant groups.^11^

We also acknowledge some limitations. First, regarding ethnic categories, although we attempted to use the same categories as the original studies, our moderator analyses required some judgements about how to combine groups. We sought to be as non-arbitrary as possible by using UK census classifications and author definitions, but clearly no scheme is definitive. This question of how to categorise groups on the meta-analytic level also applies on the study level. The authors of original studies will have had to make these decisions too and may have used a variety of criteria to do so – UK studies tended to use self-ascribed ethnicity, whereas studies in other countries classified groups by birthplace. Further, composition of apparently uniform groups differs by country, e.g. the ethnic subgroups that comprise ‘Asian’ samples. As well as these conceptual issues, when stratifying data into specific groups, there is a trade-off between aggregating and splitting groups in terms of statistical power and error control. These issues, stemming from race's social construction,^62^ make synthesising studies inescapably complicated.

In terms of exposure, rather than exclude studies that quantified group density differently, we attempted to rescale effects so that they all reflected a 10 percentage-point decrease. This allowed us to synthesise more evidence than previous reviews, but it may have resulted in imprecision and extrapolation. Additionally, the quantification of group density by geographical unit is subject to the modifiable areal unit problem.^63^

Furthermore, studies varied in how they quantified psychosis. Rather than exclude studies based on their psychosis outcome, we decided to use this as an opportunity to examine whether group density associations differ for non-clinical versus clinical outcomes. Formal moderator tests indicated some evidence that associations were stronger for the latter. This should be considered when observing differences between minority groups (also see Supplemental 7).

In terms of the evidence-base, there are broader issues of temporality and consistency, which are key criteria for assessing causation in epidemiological studies.^64,65^ Most studies were conducted in similar settings and time periods; there is a dearth of research from outside Europe, for example.^8^ Consequently, a reduced range of minority groups were included and, given that a disproportionate number of studies in the meta-analysis were conducted in the UK, generalising findings must be approached with caution.

Our review of group density associations in non-ethnic minorities was also limited by the lack of studies including such samples. This is an important priority for future research in terms of elucidating mechanisms.

Finally, most reviewed studies were cross-sectional: potential mechanisms are discussed in the next section, but there is a clear need for further longitudinal studies to identify causal pathways.^14^

### Proposed mechanisms

#### Racism and discrimination

The attenuated risk and impact of racial harassment experienced by minority groups in higher own-group neighbourhoods has been proposed as a key mechanism underpinning group density relationships.^11,30,31^ Evidence from Europe and the USA suggests that visible minorities,^48^ particularly Black individuals, are at especially high risk of experiencing discrimination and coercive pathways to psychiatric treatment.^7,11,66,67^ Evidence suggests that minorities living in lower own-group density areas also anticipate more discrimination from healthcare services.^31^ Combined with findings that ethnic minorities experience greater mental health-related stigma,^68^ this may exacerbate delays in help-seeking^69^ and has important implications for early intervention services.

Some evidence indicates that changes to neighbourhood ethnic composition can drive anti-immigration sentiment, especially in areas that have experienced rapid rates of change,^70–72^ but this has not been examined in the context of group density associations. The perceived loss of power associated with the prospect of becoming a minority has been suggested to drive majority group individuals’ exclusionary and hostile treatment of minorities.^37,73^ Consequently, some minority groups may in fact be at elevated risk of psychosis in newly high ethnic density areas. This may explain detrimental own-group density relationships observed in some populations.^30,34,48^ It is also important to contextualise studies in terms of their socio-political context, e.g. there has been a stark increase in anti-Asian discrimination during the COVID-19 pandemic.^74^ This may be an important influence in post-COVID-19 group density studies including Asian populations.

#### Deprivation

In addition to overt discrimination, disproportionate poverty or the propensity to ‘drift’ into more deprived areas were thought to be key drivers of the excess psychosis risk in ethnic minorities.^6,75,76^ However, ethnic density associations tend to persist after adjustment for deprivation.^10,16^ Furthermore, given that areas with higher density of ethnic minorities are often more deprived,^35,77^ any residual confounding might be expected to operate in the opposite direction to density effects in minority groups.^10,16^ There is, however, evidence that social drift prior to diagnosis may artifactually produce ethnic density associations in *majority* groups,^39^ which may explain between-group density effects^10^ appearing larger than within-group effects.^16^

#### Social capital

Social capital is thought to have a key role in the protective effects of own-group density.^11,36^ It has been defined as ‘connections among individuals – social networks and the norms of reciprocity and trustworthiness that arise from them’.^78^ The increased access to social capital garnered by minorities living in high own-group areas is proposed to weaken the impact of social adversity such as discrimination^11,30^ and deprivation.^79^ There is evidence that the association between social capital and psychosis risk is non-linear, with neighbourhoods characterised by high and low levels of social capital conferring the highest risk of psychosis.^26^ High social capital, particularly bonding social capital,^78^ may increase risk in individuals who experience or perceive exclusion from the networks that it represents,^80,81^ such as ethnic minorities in lower own-group density areas.^26^

#### Migration and ‘acculturation’

Studies have indicated that the stress of migration and adaptation to the host culture contribute to the excess risk of psychosis in minorities, although this risk may be reduced in those who speak the host language and have higher educational and employment prospects.^4,7^ Although we did not find moderation by country, there is some evidence to support this notion, with some studies finding lower psychosis prevalence and weaker or absent group density associations in Canada^53^ and Australia,^54^ countries where immigration policy gives preference to individuals with these characteristics.

Factors related to low ‘acculturation’ (e.g. majority language ability) are more prevalent in first-generation migrants than in their children, who are commonly more ‘assimilated’ into the host culture.^8,48,82^ Recent evidence suggests that linguistic factors confer greater risk of psychosis in first-generation migrants, whereas social disadvantage^83^ and the stress of alienation from both identities (marginalisation) or rejecting culture of origin in favour of the host culture (assimilation) are proposed to underpin risk in subsequent generations.^51,82,84^ Generational differences in the group density effect could therefore shed light on the processes driving the increased risk. However, to date literature examining this is mixed^16,48,51^ and there were too few studies stratifying by generation to allow for meaningful moderator analysis in the present review.

#### Pathways to psychosis

Both material and psychological processes likely drive group density associations, and these may not be mutually exclusive. Material processes refer to factors preventing individuals from accessing the resources and capacities required for autonomy,^85,86^ e.g. individuals who do not speak the majority language may find it harder to find work or access appropriate mental health services in low own-group density areas.^69^ This also includes deliberate attempts to exclude minority groups and restrict their access to opportunities and support networks.^66,86^ This explains why group density effects are observed in marginalised groups, including ethnic minorities,^10,16,17^ isolated single people,^18,41^ people with deprived social status^18,47^ and LGBTQ+ individuals,^12^ while there is some suggestion that minority groups with a greater share of power do not experience the same degree of risk to their mental health.^4,87^ That said, there has been limited investigation into group density associations in these groups. To identify key mechanisms, it would be theoretically useful to examine whether group density is an important social determinant of psychosis in less marginalised minority groups such as Swedish speakers in Finland, who comprise a linguistic minority but generally occupy a higher socioeconomic position and live longer than the Finnish-speaking majority.^87^

Psychological processes relate to the mental consequences of belonging to a disempowered group. There are several theoretical frameworks for conceptualising the psychological sequelae of marginalised minority group membership, including the minority stress model,^88^ social defeat^89^ and social identity theory.^90,91^ These mechanisms may be especially important in the aetiology of psychotic disorders, given that group density effects appear to have a degree of specificity to psychosis.^16,17^ Although the evidence is limited, negative evaluations of self and others^92^ (exacerbated by experiences of racism) appear to have a unique role in paranoia, but not in hallucinations.^91,93^ Supporting neurobiological evidence from non-clinical samples indicates that Black individuals in lower own-group density areas perceive greater social threat in response to White faces,^94^ suggesting a possible pathway to paranoia.^94^ Conversely, the social deafferentation hypothesis suggests that social isolation has stronger links with hallucinations.^95^ Therefore the former is perhaps a more common pathway in Black individuals and the latter in groups who experience greater linguistic and cultural barriers, e.g. first-generation migrants.^48,52,83^

These social processes highlight the importance of contextualising psychotic experiences in minority groups and considering to what extent these are understandable responses to chronic experiences of discrimination and social exclusion.^96^

### Implications

There has been limited discussion of the implications of group density findings, particularly with regard to policy. This is understandable, given that these findings could be viewed as arguments in favour of ethnic segregation. However, residential segregation has instead been associated with poorer health.^97,98^ Further, it is plausible that the risks associated with low own-group density areas are a manifestation of disempowerment experienced by that group and the effect might therefore be attenuated if minority groups experienced less social disadvantage. To appropriately address these issues, the underpinning individual-level and systemic factors must be examined.^99^

It has been argued that focusing on assimilating migrants into host cultures exacerbates the dominant culture's ‘othering’ of minority groups,^100^ creating greater disconnect between their parental and host cultures,^51^ which is likely to have unfavourable mental health consequences.^51,82^ As an alternative, strategies to create cross-cutting identities may be efficacious in increasing access to bridging social capital, which has a protective effect.^80,101^ Establishing positive intergroup contact may be especially challenging for individuals prone to psychosis, who may be more likely to perceive others as a threat,^102^ but facilitating positive contact may help foster stable social identities, in terms of minority groups’ connectedness with both their cultural group and wider community.^51^ That said, creating the social conditions to enable minorities to form strong civic identities and access bridging social capital will only be achieved by systemic changes to reduce community-level social inequality and, crucially, the structural racism that sustains inequities in the social, economic and living circumstances of minority groups.^7,99^

As well as these wider systemic issues, useful targets for clinical intervention might include strategies to improve clinicians’ cultural competence^99,103^ and understanding of the disempowerment experienced by minority groups, and how this may be amplified in low own-group density areas. To better inform interventions, further investigation is needed to determine when in life low-own group density confers the greatest risk.^36^ Therapeutic approaches that aim to develop strong social identities might also be efficacious.

### Future research

The logic of group density designs assumes that individuals living in low and high own-group density areas can be straightforwardly compared.^14^ Given that the reasons for large minority group populations in particular areas are not arbitrary – rather, they are linked with factors such as family, housing cost and employment^4^ – it is difficult to disentangle the contextual and compositional effects^104^ of own-group density. There is a clear need for longitudinal designs^17^ and demonstrations that associations persist across different settings and time periods.^4^

The present review suggests that the group density effect is complex and appears to vary by minority group, with the strongest associations observed in Black populations. To substantiate our findings and elucidate mechanisms, more studies examining specific ethnic minorities are required. Future work should also test for group density associations in minorities defined by other characteristics. In addition to epidemiological studies, proposed avenues for future research should be explored using different methodologies, such as qualitative interviews,^105^ experience-based approaches,^106^ neurobiological studies^94^ and VR^44^ to better capture the subjective experiences driving group density effects.^8^

## Data Availability

Data availability is not applicable to this article as no new data were created or analysed in this study.

## Supplementary material

For supplementary material accompanying this paper visit http://dx.doi.org/10.1192/bjp.2021.96. click here to view supplementary material
