# Supplementary material for: The ethnic density effect in psychosis: a systematic review and multilevel meta-analysis
Source: Br J Psychiatry. 2021 Dec;219(6):632–43. doi: 10.1192/bjp.2021.96 (PMC8636614; doi:10.1192/bjp.2021.96)
Supplement: Supplementary file 1 [file S0007125021000969sup.zip › S0007125021000969sup001.docx]

**Table 1.** Characteristics of the included studies (*n*=32), meta-analysis (*n*=10)

| **Author & setting** | **Datasets & time period** | **Minority group classification** | **Psychosis outcome** | **Geographic unit (*n* areas/av. pop.)** | **Group density exposure** | **Individual & area-level covariates** | **Statistical analysis** | **Minority groups (cases/total minority group sample)** | **Results** |
| --- | --- | --- | --- | --- | --- | --- | --- | --- | --- |
| Anglin, Lui, Schneider & Ellman, (2020)^52^  Northeastern USA | Public university system. Students recruited via a participant recruitment website.  Time period: 2011-2016 | Self-ascribed Black/African American/African descent or as a 1^st^ or 2^nd^ gen immigrant, answers grouped into USA census categories. | The positive subscale of the Prodromal Questionnaire-Likert [PQ-Likert] | Neighbourhood (NA) | Perceived racial composition of neighbourhood prior to age 12 and after age 12 (*e.g.,* mostly Black, mostly Latino, mostly Asian…) | Individual [I]: Immigrant status, poverty index, age, racial & ethnic group, & lifetime cannabis use  Area [A]: None | ANCOVA, post-hoc Bonferroni-corrected t-tests | Combined (NR/1330)  Black (NR/427)  Hispanic/Latino (NR/429)  Asian (NR/474)  1^st^ generation migrants (NR/560)  2^nd^ generation migrants (NR/657)  Non-immigrant (NR/112)  Mean no. of psychotic experiences endorsed=12.25 | For both before and after age 12, the highest psychotic experiences were reported by ethnic & racial minorities who recounted living in racially discordant neighbourhoods compared to minorities who grew up in racially concordant, mixed, or majority White areas. Ethnic minority individuals who perceived change in the racial composition of their neighbourhood after age 12 reported more psychotic experiences than those who perceived no change - in stratified analysis, this was only sig. in the Black group [*F*_1,425_=5.08, *p*=.025], |
| **Bécares, Nazroo & Stafford (2009)**  England & Wales, UK | Fourth National Survey on Ethnic Minorities [FNS] & 1991 UK Census.  Time period: 1994 (1 year). | Self-ascribed | Psychosis Screening Questionnaire [PSQ] | Electoral ward [EW] (9527, 5327) | 10% increase | I: Age, sex, and socioeconomic position  A: Deprivation | Multiple logistic regression | Combined (NR/4277)  Black Caribbean (NR/1215)  Indian (NR/1278)  Pakistani (NR/1190)  Bangladeshi (NR/594) | OR=0.99  OR=0.83  OR=0.90  OR=1.44 [SS]  OR=0.81 |
| Bécares & Das-Munshi (2013)  **Das-Munshi, Bécares, Boydell et al., (2012)**  England, UK | Ethnic Minority Psychiatric Illness Rates in the Community [EMPIRIC], merged dataset of the English samples from the 2005 and 2007 Citizenship Survey [CS] & 2001 UK census.  Time period: 2005 & 2007 (2 years). | Self-ascribed  (Irish – place of birth or parent’s place of birth) | PSQ | Middle Super Output Area [MSOA]  (7193, 7200) | 10% decrease | I: Age, sex, social class, marital status, education, and generational status  A: Deprivation  *n.b. only 2013 study adjusted for generational status* | Multiple logistic regression | Combined (305/3444)  Irish (59/733)  Black Caribbean (83/694)  Indian (58/643)  Pakistani (72/724)  Bangladeshi (33/650)  White British (majority)  (50/837) | OR=1.07 [SS]  OR=5.44  OR=1.05  OR=1.38 [SS]  OR=1.17  OR=1.26  OR=0.91  *n.b., effect sizes from 2012 study* |
| Bhavsar, Boydell, Murray & Power (2014)  Lambeth, South London, UK | Lambeth Early Onset [LEO] case register & 2001 UK census.  Time period: January 2000 – December 2007 (8 years). | NR | Schizophrenia [SZ] first incident cases [RDC criteria] | EW (21, NR) | 100% increase | I: Age & sex  A: Deprivation | Multilevel Poisson regression | Black Caribbean (NR)  Black African (NR) | IRR= 0.003 [SS]  IRR=0.04 |
| Boydell, van Os, McKenzie et al., (2001)  Camberwell, South London, UK | Bethlem Royal & Maudsley NHS Trust & 1991 UK census  Time period: 1988 – 1997 (9 years) | Any self-ascribed ethnicity other than White  (if not available, place of birth, parents place of birth, and any description of colour) | SZ first incident cases [RDC criteria, ICD-9 & ICD-10] | EW (15, 10,000) | Thirds of non-white ethnic density | I: Age & sex  A: Deprivation | Multilevel Poisson regression | Non-white ethnic minority (126/NR)– comprising Black Caribbean, Black African, and other | Lowest third (8-22.8%)  IRR=4.40 [SS]  Middle third (23-28.1%)  IRR=3.63 [SS]  Highest third (28.2-57%)  IRR=2.38 [SS] |
| Das-Munshi, Schofield, Bhavsar et al., (2019)  Lambeth, Lewisham, Croydon, & Southwark, South London, UK | South London & Maudsley NHS Trust Clinical Record Interactive Search [CRIS] system & 2011 UK census  Time period: January 2007 – December 2014 (8 years) | Self-ascribed ethnicity grouped using UK Office for National Statistics [ONS] ethnic group classifications | All-, natural, and unnatural-cause mortality in individuals with ICD-10 SMI diagnoses (schizophrenia-spectrum disorders (F2*) and bipolar disorders (F30 and F31) | Lower Super Output Area [LSOA] (NR/1614) | Highest and lowest own minority group density | I: Age, sex, diagnosis, marital status, substance use disorders, ethnicity*own ethnic density interaction  A: Deprivation, urbanicity, & social fragmentation | Multilevel Poisson regression | Combined (637/9154)  Black African (106/2510)  Black Caribbean (332/4840)  South Asian (95/1256)  Irish (104/548)  White British (majority) (1130/9047)  *n.b., above refer to all-cause deaths/sample* | IRR=0.96, IRR=0.52 [SS]  Interaction: *p*=0.036 [SS]  IRR=0.79, IRR=0.25 [SS]  Interaction: *p*=0.068  IRR=0.70 [SS], IRR=0.58 [SS]  Interaction: *p*=0.62  IRR=1.08, IRR=0.07 [SS]  Interaction: *p*=0.015 [SS]  IRR=0.97, IRR=1.80  Interaction: *p*=0.65  REF  *n.b., 1^st^ IRR =lowest own-group density (0%), 2^nd^ IRR=highest ethnic density (95% for combined, 50%, 30%, 90%, & 11% respectively for specific minority groups). Data for specific groups taken from paper’s supplementary material* |
| **Dykxhoorn, Lewis, Hollander, Kirkbride & Dalman, (2020)**  Sweden | Register of the Total Population, the immigration and emigration register (STATIV), the Multi-Generation register, and the National Patient Register. (Linked by Psychiatry Sweden)  Time period: January 1982 – December 2016 (35 years) | Place of birth & parents place of birth (born outside of Sweden: 1^st^ gen, born in Sweden, at least one parent born outside of Sweden: 2^nd^ gen) | NAP (F20-29) [ICD-10] | Small Areas for Market Statistics [SAMS]  (9208/726) | 5% decrease & effect at different quintiles (highest=REF)  *n.b., migrant density exposure measured at age 15 or after immigration to Sweden* | I: Age, sex, calendar year, generation status, lone dwelling, time since migration, family disposable income, receipt of social welfare & family unemployment A: Population density, proportion of lone dwelling households & deprivation | Multilevel Cox proportional  hazards regression | Nordic 1^st^ gen (103/131882)  2^nd^ gen (644/766149  European 1^st^ gen (693/880211)  2^nd^ gen (270/310934)  Asian 1^st^ gen (297/365971)  2^nd^ gen (61/92699)  Middle Eastern & North African 1^st^ gen (693/796928)  2^nd^ gen (349/471962)  Sub-Saharan African 1^st^ gen (550/261899)  2^nd^ gen (127/72516)  North American 1^st^ gen (50/55558)  2^nd^ gen (6/6338)  South American 1^st^ gen (79/102857)  2^nd^ gen (66/84025)  Swedish migrant 2^nd^ gen (390/456995)  Mixed migrant 2^nd^ gen  (201/153726)  *n.b., second value refer to person-years* | HR=1.01  HR=0.97  HR=0.98  HR=1.06  HR=1.42 [SS]  HR=1.15  HR=1.03  HR=1.00  HR=1.28 [SS]  HR=0.94  HR=1.77  HR=0.89  HR=0.67  HR=1.15  HR=0.83  HR=1.17  Combined migrant density:  Quintile 1 (lowest) HR=1.36 [SS]; Q2 HR=1.14 [SS]; Q3 HR=1.11; Q4 HR=1.07; Q5 (highest)=REF |
| Eilbracht, Stevens, Wigman, van Dorsselaer & Vollebergh (2015)  The Netherlands | Dutch Health Behaviour in School-Aged Children [HBSC]  Time period: 2005 (<1 year) | Father, mother, or both parents born in a non-Western country | The Community Assessment of Psychotic Experiences [CAPE] positive experiences subscale | Classroom  (NR, 21) | Assoc. between class proportion of minority group pupils & PEs | I: Age, sex, education, family wealth  A: Class size | Multilevel multivariate regression | Combined (NR/769)  Moroccan (NR/228)  Turkish (NR/182)  Surinamese or Antillean (NR/178)  Other non-Western (NR/181)  Dutch (majority) (NR/3606) | Sig. increase in paranoia with increasing ethnic density in the Dutch majority pupils (*b*= 0.16, *p*<0.05) but no sig. effects in the combined minority group (*b*= -0.05, *p*=0.99) or for specific minority groups (data NR). No sig. ethnic density effects for any other PEs (AVHs, delusions, grandiosity, or paranormal beliefs) |
| Halpern & Nazroo (2000)  England & Wales, UK | Policy Studies Institute [PSI] National Community Survey in England & Wales 1993/94 & 1991 UK census  Time period: 1993 – 1994 (1 year) | Self-ascribed | PSQ | EW (NR) | Correlation between own-group density and subclinical psychotic symptoms | I: Age, sex, hardship, migration & language  A: None | Multivariate linear regression | Combined (5226/5196)  Black Caribbean (1215/1205)  Indian (1278/1273)  African Asian (733/728)  Pakistani (1190/1185)  Bangladeshi (594/591)  Chinese (216/214)  White (majority) (NR/2867)  *n.b., 1^st^ value refers to number of PSQ symptoms reported, 2^nd^ value is the sample size.* | *b*= -.071, *p*<0.001 [SS]  *b*= -.058, *p*<0.05 [SS]  *b*= -.126, *p*<0.001 [SS]  *b*=-.043  *b*=.043  *b*= -.140, *p<*0.001 [SS]  *b*=-.044  *b*= -.040, *p*<0.05 [SS] |
| Heslin, Khondoker, Shetty et al., (2018)  Lambeth, Lewisham, Croydon, & Southwark, South London, UK | South London & Maudsley NHS Trust Clinical Record Interactive Search [CRIS] system & 2011 UK census  Time period: January 2007 – December 2010 (~4 years) | Ethnicity recorded in patient records according to UK ONS ethnic group classifications | Inpatient days following an ICD-10 diagnosis of any psychotic disorder | LSOA (NR, 1500) | Regression of inpatient days over 5 years and overall ethnic density | I: Length of time with service, age, sex, & BME status | Negative binomial regression | Combined (NR/1515),  Black African (NR/430),  Black Other (NR/228),  Black Caribbean (NR/209),  Indian (NR/32), Pakistani (NR/25),  Chinese (NR/21), Bangladeshi, (NR/14), Other Asian (NR/95),  Irish (NR/48), White & Black, Caribbean (NR/20), White & Black African (NR/12), White & Asian (NR/7), Other mixed (NR/12),  Any other ethnic group (NR/161), White other (NR/201), White (majority) (NR/632) | *b=*0.59 (overall ethnic density)  Overall ethnic density was not associated with days as an inpatient |
| Horrevorts, Monshouwer, Wigman & Vollebergh (2014)  The Netherlands | The Dutch health behaviour in school-aged children survey [HSBC]  October – November 2005 (<1 year) | Self-ascribed. Children were asked if they had bullied or been a victim of bullying in the past 2 months. | CAPE | Classroom (NR) | Interaction between classroom-level bullying status x bully climate, continuous measure of group density | None | Multilevel regression | Bully (NR/333)  Victim (NR/216)  Bully-victim (NR/55)  Non-involved (NR/3978)  Mean scores on subclinical psychotic experiences (CAPE):  Bully=1.45  Victim=1.53  Bully-victim=1.59  Non-involved=1.34 | Bully climate x bully  *b*=-0.002  Bully climate x victim  *b*=-0.004 [SS]  Bully climate x bully-victim  *b*=-0.006  The association between bully-victim status and subclinical psychosis was attenuated in classes with higher bully climate |
|  |  |  |  |  |  |  |  |  |  |
| Kirkbride, Morgan, Fearon, Dazzan, Murray & Jones (2007)  Kirkbride, Boydell, Ploubidis et al., (2008)  Lambeth & two-thirds of Southwark, South London, UK | Aetiology and Ethnicity in Schizophrenia and Other Psychoses [AESOP] study & 2001 UK census  Time period: September 1997 – August 1999 (2 years) | Self-ascribed, place of birth, & parents place of birth, grouped using UK ONS ethnic group classifications | First incident cases of SZ and other non-affective psychoses [NAP]  [ICD-10] | Census Area Statistic [CAS] wards (33/5880) | 1% increase & between-groups at each third of combined minority group density | I: Age, sex  A: Area-level variables not included in model  *n.b., in Kirkbride et al., (2008) voter turnout replaced with social cohesion & trust and social disorganisation* | Multilevel  Poisson regression | Combined (163/201720)  Black Caribbean (NR)  Black African (NR)  Asian (NR)  Mixed ethnicity (NR)  White other (NR)  Other ethnicity (NR)  White British (majority) (55/363856)  *n.b., 2^nd^ value refers to person-years* | Within groups:  IRR=1.00 (SZ)  Interaction: *p*=0.19  IRR=1.04 (Other NAP)  Interaction: *p*=0.43  Between groups:  Lowest third (24.8-47.1%) IRR=6.50  Middle third (47.2-56.1%) IRR=2.13  Upper third (56.4-74.3%) IRR=3.81  *n.b., effect sizes from 2007 study* |
| Kirkbride, Jones, Ullrich, & Coid (2014)  City & Hackney, Newham, & Tower Hamlets, East London, UK | The East London first-episode psychosis [ELFEP] study & 2001 UK census  Time period: December 1996 – November 1998 (2 years – City & Hackney)  December 1998 -November 2000 (2 years – Newham & Tower Hamlets) | Self-ascribed, place of birth, & parents place of birth, grouped using UK ONS ethnic group classifications | First incident cases of NAP [DSM-IV] | Super Output Area [SOA]  (56, 6195) | 1-SD increase of own-group density | I: Age, sex, social class  A: None | Bayesian Hierarchical Modelling | Black African (49/NR)  Black Caribbean (55/NR)  Bangladeshi (53/NR)  Non-British White (38/NR)  White British (majority) (68/NR) | RR=0.70 [SS]  NR  NR  NR  *n.b., RR shows effect for NAP. Data NR but associations not sig. for Black Caribbean, Bangladeshi, & non-British White groups* |
| **Menezes, Georgiades, & Boyle (2011)**  Canada | Canadian Community Health Survey [CCHS] & 2001 Canadian census  Time period: 2002 (1 year) | Immigrant status assigned if individual was born outside Canada and not born a Canadian citizen | Self-reported lifetime prevalence [LTP] of SZ | Dissemination area [DA] (8145, 400-700) | Immigrant status x immigrant concentration | I: Age, sex, income, marital status, education  A: Disadvantage (% with low income, rentals, & that moved in the last year) | Multilevel logistic regression | Immigrant status (born in Asia, Oceania, or Europe) (31/7784) | Immigrant status x concentration interaction:  OR=0.81  *n.b., lower SZ in immigrants. Additional protective effect of migrant group density but this was not sig.* |
| Mezuk, Li, Cederin et al., (2015)  Stockholm, Gothenburg, & Malmö, Sweden | Nationwide psychiatric inpatient and outpatient registries. & 2005 Swedish census  Time period: 2005 - 2010 (5 years) | Place of birth & parents place of birth (born outside of Sweden: 1^st^ gen, born in Sweden, at least one parent born outside of Sweden: 2^nd^ gen) | NAP & AP first incident cases [ICD-10] | SAMS (1490, 1000) | Psychosis risk living in an ethnic enclave compared to majority Swedish area | I: Age, sex, education, income, & 1^st^ or 2^nd^ gen status  A: Deprivation (% with low educational attainment, low income, unemployed, & receiving social welfare) | Multilevel logistic regression | Iraqi (NR/19975)  Other (NR/232356)  Swedish-born (majority) (NR/698648)  *n.b., ‘Other’ group comprised migrants from Finland, Asia (excluding Turkey, Iran or Iraq), countries in Africa, former Yugoslavia, Iran, Poland, Turkey, Bosnia, Chile, and other nations in South America (other than Chile)* | OR=1.66 [NAP], OR=1.04 [AP]  OR=0.93 [NAP], OR=0.93[AP]  OR=1.36 [NAP], OR=1.12 [AP] [SS]  *n.b., results for broadly defined AP & NAP* |
| O’Donoghue, Yung, Wood et al., (2015)  Melbourne, Australia | The Personal Assessment and Crisis Evaluation [PACE] clinic & 2001 Australian census  Time period: 2000-2006 (6 years) | Place of birth & parents place of birth (born outside of Australia: 1^st^ gen, born in Australia, at least one parent born outside of Australia: 2^nd^ gen) | Meet criteria for at least one of the three Ultra High Risk [UHR] groups | Postcode Area [PA] (57, 13527) | Quartiles of ethnic density (highest=REF) | I: None  A: Social deprivation | Poisson regression | Total migrants (59/NR)  1^st^ generation migrants (10/NR)  2^nd^ generation migrants (49/NR) | Total migrants:  Low (7.7-23.2%)  IRR=0.77  Below av. (24.4-32.5%) IRR=1.46  Above av. (32.7-40%) IRR=1.86  High (42-50.9%) [REF]  1^st^ generation migrants:  Low (7.7-23.2%) IRR=0.72  Below av. (24.4-32.5%) IRR=0.93  Above av. (32.7-40%) IRR=1.29  High (42-50.9%) [REF]  *n.b. above data from supplementary material (adjusted for deprivation). Percentages for migrant density quartiles provided by author.* |
| **Richardson, Hameed, Perez, Jones & Kirkbride (2018)**  East Anglia, England, UK | Social Epidemiology of Psychoses in East Anglia [SEPEA] study & 2011 UK census  Time period: August 2009 – February 2013 (3.5 years) | Self-ascribed ethnicity grouped using UK ONS ethnic group classifications | NAP & AP first incident cases [ICD-10] | Statistical Ward [SW] (530, 3992) | 1% increase | I: Age, sex, socioeconomic status, ethnicity  A: Deprivation, urbanicity, & social isolation (all psychoses). Ethnic diversity, deprivation, social isolation (NAP). Ethnic fragmentation (AP) | Multilevel Poisson regression | Combined (160/398511),  Black African (21 NAP, 1 AP/ 17193), Black Caribbean (6 NAP, 3 AP/5973), Mixed white & black Caribbean (5 NAP, 2 AP/13100), Mixed Other (11 NAP, 6 AP/30927), Indian (2 NAP, 0 AP/27911), Pakistani (13 NAP, 3 AP/20126), Bangladeshi (5 NAP, 1 AP/8403), Arab (4 NAP, 0 AP/4838), Other ethnic group (13 NAP, 2 AP/62875), White Other 50 NAP, 12 AP/207165), White British (majority) (418 NAP, 53 AP/1623285)  *n.b., 2^nd^ value refers to person-years* | OR=1.00 (all psychoses)  OR=0.99 (NAP)  OR=0.98 (AP) [SS] |
| **Schofield, Ashworth & Jones (2011)**  Lambeth, South London, UK | Electronic GP patient records (the Lambeth DataNet) & 2001 UK census  Time period: January 1996 – November 2006 (10 years) | Patient ethnicity codes were grouped using UK ONS ethnic group classifications (Black or Black British) | Psychosis first incident cases [DSM-IV] | LSOA (NR, 1500)  CAS wards (NR, 6000) | High & low ethnic density. Associations at quintiles of ethnic density (highest=REF) | I: Age & sex  A: Deprivation | Multilevel Poisson regression | Black (109/23693)  White British (majority) (87/37278) | Between groups (LSOA):  High (25-62%) IRR=1.48  Low (0-24%) IRR=2.88 [SS]  Within groups (LSOA):  Highest (43%) [REF]  High (31%) IRR=2.50 [SS]  Mid (24%) IRR=3.59 [SS]  Low (19%) IRR=5.39 [SS]  Lowest (11%) IRR=5.24 [SS]  Within groups (CAS ward):  Highest (37%) [REF]  High (30%) IRR=1.06  Mid (26%) IRR=1.63  Low (22%) IRR=1.90  Lowest (11%) IRR=1.14  *n.b., CAS ward data provided by author. Within groups model at CAS level was non-sig. The same within-groups models for White British sample were non-sig.* |
| **Schofield, Das-Munshi, Bécares et al., (2016)**  Lambeth & Southwark, South London, UK | South East London Community Health [SELCoH] study: 2008-2010 & 2011 UK census  Time period: 2008-2010 (2 years) | Self-ascribed Black Caribbean or Black African from UK ONS ethnic group classifications | PSQ | LSOA (322,1500) | 10% decrease | I: Age & sex  A: Deprivation | Multilevel logistic regression | Black combined (98/377)  Black African (NR/234)  Black Caribbean (NR/143)  Single household status (51/212)  Disadvantaged social class (101/421) | OR=1.34 [SS]  OR=1.15  OR=1.99  OR=2.18  OR=0.88  *n.b., effects for Black African & Black Caribbean are from paper’s supplementary material* |
| **Schofield, Thygesen, Das-Munshi et al., (2017)**  Schofield, Thygesen, Das-Munshi et al., (2018)  Denmark | Danish Civil Registration System dataset, the Danish Psychiatric  Central Register, & Integrated Database for Longitudinal Labour  Market Research  Time period: residents born between January 1965 & December 1997 & residing in Denmark on their 15^th^ Birthday followed up until July 2013 (~33 years) | Place of birth & parents place of birth (born outside of Denmark: 1^st^ gen, born in Denmark, both parents born outside of Denmark: 2^nd^ gen) | NAP first incident cases [ICD-10] | Parish units (1167, 3564) | Quintiles of ethnic density (highest=REF)  *n.b. variables measured at age 15* | I: Age, sex, calendar period, parental psychiatric history & income  A: Urbanicity | Multilevel Poisson regression | African (362/13118) 1^st^ gen (236/7187)  2^nd^ gen (80/4593)  Non-Scandinavian European (1175/58939) 1^st^ gen (585/24436) 2^nd^ gen (410/25984)  Asian (415/24512)  Middle Eastern (529/28762)  1^st^ gen (412/17983) 2^nd^ gen (102/10293)  Danish (majority) (24410/1921874) | Lowest (<0.4%) IRR=1.94, low (0.4-0.9%) IRR=2.17, mid (0.9-1.7%) IRR=1.11, high (1.7-3.7%) IRR=1.20, highest (3.7-18.5%) [REF]  Lowest (<2.3%) IRR=1.99, low (2.3-3.9%) IRR=1.60, mid (3.9-5.9%) IRR=1.39, high (5.9-9.4%) IRR=1.43, highest (9.4-26.4%) [REF]  Lowest (<0.6%) IRR=1.63, low (0.6-1.2) IRR=1.00, mid (1.2-2.1%) IRR=0.93, high (2.1-3.9%) IRR=1.00, highest (3.9-14.3%) [REF]  Lowest (<0.8) IRR=1.68, low (0.8-1.7%) IRR=1.29, mid (1.7-3.3%) IRR=1.04, high (3.3-6.7%) IRR=1.23, highest (6.7-40%) [REF]  *n.b. sig differences between highest and lowest quintiles for all minority groups, strongest for African sample.*  Generational differences (Schofield, Thygesen, Das-Munshi et al., 2018):  African IRR=1.33 [SS],  Non-Scandinavian European IRR=1.08 [SS],  Middle Eastern IRR=1.25 [SS] |
| Stouten, Veling, Laan, & Van der Gaag (2016)  The Hague, the Netherlands | Centre for Early Psychosis referrals & the Central Bureau of Statistics, the Netherlands  Time period: December 2009 – December 2012 (3 years, 1 month) | Place of birth & parents place of birth (born outside of the Netherlands: 1^st^ gen, born in the Netherlands, at least one parent born outside of the Netherlands: 2^nd^ gen) | NAP first incident cases [DSM-IV], symptom dimensions & remission [PANSS & SCI-SR] | Neighbourhoods (44, max. 38000) | Baseline ethnic density as a predictor of symptomatic outcomes at 12 months follow up | I: Age, income & education  A: None | Backwards regression models | 1^st^ gen (60/NR)  2^nd^ gen (56/NR)  (Comprising migrants from Morocco, the Netherlands Antilles, Surinam, Turkey, other Western & other non-Western countries) | Baseline ethnic density was not a sig. predictor of any psychosis outcome at follow up. |
| Terhune, Dykxhoorn, Mackay, Hollander & Kirkbride (2020)  Sweden | Psychiatry Sweden anonymised database of linked national registers  1985 – December 2016 (~32 years) | Place of birth & parents place of birth (born outside of Sweden: 1^st^ gen, born in Sweden, at least one parent born outside of Sweden: 2^nd^ gen) | Compulsory admission status at the time of the first diagnosis of psychotic disorder. | SAMS (7416/ 1000-2000) | 1-SD increase | I: Age, sex, region of origin  A: Population density  (no other variables improved the final model fit) | Multilevel logistic regression | Combined (1800 1^st^ gen, 2605 2^nd^ gen)  European 1^st^ gen  2^nd^ gen  Asian & Oceanic 1^st^ gen  2^nd^ gen  Middle Eastern & North African 1^st^ gen  2^nd^ gen  Sub-Saharan African 1^st^ gen  2^nd^ gen  North & South American 1^st^ gen  2^nd^ gen  Swedish-Nordic 2^nd^ gen  Swedish-migrant 2^nd^ gen  Mixed Migrant 2^nd^ gen | OR=1.12 [SS]  OR=1.25  OR=2.19  OR=1.24  OR=1.87  OR=1.45  OR=2.22  OR=1.99  OR=3.59  OR=1.47  OR=2.31  OR=2.41  OR=2.10  OR=1.54 |
| Termorshuizen, Smeets, Braam & Veling (2014)  Utrecht, the Netherlands | Psychiatric Case Register Middle Netherlands [PCR-MN] & Dutch population registry (Central Bureau of Statistics, the Netherlands)  Time period: January 2000 -December 2009 (9 years) | Assigned by country of birth of parent(s) born outside of the Netherlands. If both parents were born in different countries, maternal country of birth used. | NAP first incident cases [DSM-IV] | Districts (10, 27525)  Neighbourhoods (98, 2808) | Thirds of ethnic density  (lowest=REF) | I: Age & sex  A: Socioeconomic status (mean income of area) | Multilevel Poisson regression | Turkish (81/12309)  Moroccan (222/21409)  Surinamese/Antillean (155/13404)  Other non-Western (127/20230)  Dutch (majority) (1242/284747) | Between groups [SS]: Low (<6.5%) RR=1.39, mid (6.5–9.6 %) RR=1.19, high (>9.6%) RR=0.78. Within groups: Low [REF], mid RR=0.97, high RR=0.63  Between groups [SS]: Low (<11.3 %) RR=2.18, mid (11.3–27.6%) RR=1.79, high (>27.6) RR=1.01. Within groups: Low [REF], mid RR=1.02, high=0.64  Between groups [SS] Low (<3.2 %) RR=3.44, mid (3.2–4.9%) RR=2.32, high (>4.9%) RR=2.12. Within groups [SS]: Low [REF], mid RR=0.91, high RR=0.51  Between groups [SS] Low (<3.5 %) RR=2.74, mid (3.5–6.3%) RR=2.09,  high (>6.3%) RR=1.35. Within groups: Low [REF], mid RR=0.88, high RR=0.67  Sig. increased NAP risk in Dutch majority group with increasing overall ‘Non-Western’ minorities and ‘Other Non-Western’ group at the neighbourhood level  *n.b., for combined ‘all non-Western minority group’, sig. between-group effects found at the neighbourhood and district level but effects not sig. for within-groups analyses* |
| **Termorshuizen, Heerdink & Selten (2018)**  Amsterdam, Rotterdam, the Hague, & Utrecht, the Netherlands | The Health Care Institute Netherlands & & Dutch population registry (Central Bureau of Statistics, the Netherlands)  Time period: 2013 (1 year) | Assigned by country of birth of parent(s) born outside of the Netherlands. If both parents were born in different countries, maternal country of birth used. | Dispensed anti-psychotic medication [ATC code N05A, including N05AN01, Lithium] | Neighbourhoods (NR/2808) | Quintiles of ethnic density (lowest=REF) | I: Age, sex, & household composition  A: Socioeconomic status (At least 138 per 1000 households in a neighbourhood are dependent on the social welfare system) | Multivariable logistic regression | Turkish (3775/105460)  Moroccan (5207/115455)  Surinamese (4252/147123)  Antillean (949/41430)  Dutch (majority) (21918/1043732) | Lowest (<4.9%) [REF], low (4.9–9.4%) OR=1.15, mid (9.4-14%) OR=1.16, high (14-22.5%) OR=1.10, highest (>22.5%) OR=1.05  p=0.0375 [SS]  Lowest (<5.7) [REF], low (5.7-10.7%) OR=0.99, mid (10.7-15.8%) OR=1.05, high (15.8-22.1%) OR=0.96, highest (>22.1%) OR=0.93  p=0.0777  Lowest (<5.5%) [REF], low (5.5-8.9%) OR=0.84, mid (8.9-11.8%) OR=0.86, high (11.8-18.9%) OR=0.85, highest (>18.9%) OR=0.64 p<0.001[SS]  Lowest (<1.1%) [REF], low (1.1-2.3%) OR=0.90, mid (2.3-4%) OR=0.73, high (4-5.9%) OR=0.64, highest (>5.9%) OR=0.49  p<0.001 [SS]  Sig. increase in antipsychotic use in Dutch majority with increasing minority group density (for all minority groups) |
| van Os, Driessen, Gunther & Delespaul (2000)  Maastricht, the Netherlands | Maastricht Mental Health Case Register [MHCR] & Municipal authority register  Time period: 1986-1997 (11 years) | Marital status reported in the MHCR | SZ/related disorders incident cases [ICD-9] | Neighbourhoods (35/2804) | Interaction between single marital status x neighbourhood where the proportion of others living alone is below the city-level mean | I: Age, gender, marital status, age-by-gender interaction and marital-status-by-gender interaction.  A: None | Multilevel Poisson regression | Single (141/NR) | RR=10.33, p<0.001 [SS] |
| Veling, Susser, van Os, Mackenbach, Selten & Hoek (2008)  The Hague, the Netherlands | Cases ascertained by Psychiatric residents at the early psychosis department & The Hague municipal population register  Time period: 1997-1999, 2000-2007 (7 years) | Assigned by country of birth of parent(s) born outside of the Netherlands. If both parents were born in different countries, maternal country of birth used | First incident cases of a psychotic disorder [DSM-IV] | Neighbourhoods (44, max. 38000) | High and low ethnic density | I: Age, sex, & marital status  A: Socioeconomic level | Multilevel Poisson regression | Combined (240/413586)  Moroccan (91/88249)  Surinamese (94/203088)  Turkish (55/122249)  Dutch (majority) (226/1056172)  *n.b., second value refers to person-years* | Low IRR=2.36, high IRR=1.25  Continuous: IRR=0.95, *p*=0.0001 [SS]  Low IRR=4.43, high IRR=1.56  Continuous: IRR=0.93*, p*=0.002 [SS]  Low IRR=1.88, high IRR=1.19  Continuous: IRR=0.98*, p*=0.334  Low IRR=1.74, high IRR=1.12  Continuous: IRR=0.97, *p*=0.109  REF  *n.b., effect sizes for ethnicity stratified as two highest ethnic density neighbourhoods and all other neighbourhoods* |
| Veling, Brinkman, Dorrestijn & van der Gaag (2014)  The Hague, the Netherlands | Participants with FEP [DSM-IV] recruited from a specialist service for early psychosis in the Hague; Controls recruited from Delft University of Technology staff and students  Time period: NR | NR | Green Paranoid Thoughts Scale, the Social Interaction Anxiety Scale, the Davos Assessment of Cognitive Biases Scale, the Self-Esteem Rating Scale, distance from avatars, Heart rate & galvanic skin response, subjective distress, & State Social Paranoia scale | VR café | 95% of the avatars appeared White European or 75% North African depending on ethnic background of the participant. | NR | Nonparametric tests and linear mixed model analyses | Non-Dutch origin (11 FEP)  Dutch origin (majority) (6 FEP, 24 controls) | Sig. higher galvanic skin response in FEP participants in ‘other’ compared to ‘own’ ethnicity condition [*F*_1.32_ =9.82, *p*=0.004]. This was not observed in controls.  Overall, participants positioned themselves sig. further away from avatars in the ‘other’ ethnicity condition, but only in the low population density environments [*F*_1.39_ =5.08, *p*=0.030].  No sig. effects for other outcomes. |
| Veling, Pot-Kolder, Counotte, van Os & van der Gaag (2016)  The Hague, the Netherlands | UHR [CAARMS], FEP [DSM-IV], and sibling groups were recruited from five psychiatric institutes. Controls were recruited via flyers which were distributed to schools, dentist offices, and Psychiatric institutes in the Hague  Time period: NR | NR | Green Paranoid Thoughts Scale, Social Interaction  Anxiety Scale, Community Assessment of Psychic Experiences, distance from avatars, subjective distress, & State Social Paranoia Scale | VR bar | 80% of the avatars appeared Dutch or North African depending on ethnic background of the participant. | I: Age, sex, education & psychosis liability  A: NA | Chi square tests, ANOVA & multilevel random intercept regression models | Non-Dutch origin (16 controls, 11 siblings, 5 UHR, 26 FEP)  Dutch origin (majority) (37 controls, 31 siblings, 15 UHR, 29 psychosis) | Compared with participants with low psychosis liability, individuals with high psychosis liability had sig. higher paranoia [*b=*3.62 (95% CI 1.39-5.84)] and distress [*b=*17.94 (95% CI 10.99-24.90)] in response to social stress in VR.  However, no sig. association between ethnic density and either outcome. |
| **Zammit, Lewis, Rasbash, Dalman, Gustafsson & Allebeck (2010)**  Sweden | The Swedish National Patient Register, Multi- Generation Register, National Schools register, & the Swedish Census  Time period: residents born between January 1972 & December 1977 & residing in Sweden on their 16^th^ Birthday followed up until December 2013 (~31 years) | Being foreign-born, Deprived status (parents unemployed/on benefits/low income), Social fragmentation (single parent family/moved municipality/immigrated during childhood), Low grade (lower than av. School-level grade) | Cases of a psychotic disorder [ICD-8:10] | School (1264, 161) | 10% increase | I: Foreign-born, social fragmentation, grade  A: As above & variance components at school, municipality & county levels | Multilevel logistic regression | Foreign-born (NR)  Deprived status (NR)  Social fragmentation (NR)  Low grade (NR)  Total sample (328 SZ, 741 other NAP, 355 AP, 953 Other psychoses/203829) | OR=0.95 [SS]  Interaction: *p*=0.016  OR=0.92  Interaction: *p*=0.057  OR=0.92 [SS]  Interaction: *p*=0.004  OR=1.04  Interaction: *p*=0.554  *n.b., above are interaction effects for ‘any psychosis’. Effects broken down by SZ, NAP, AP & ‘Other’ psychoses can be found in the paper’s supplementary material* |

*n.b., studies in* ***bold*** *contributed data to the meta-analysis*
