# Supplementary material for: The ethnic density effect in psychosis: a systematic review and multilevel meta-analysis
Source: Br J Psychiatry. 2021 Dec;219(6):632–43. doi: 10.1192/bjp.2021.96 (PMC8636614; doi:10.1192/bjp.2021.96)
Supplement: Supplementary file 1 [file S0007125021000969sup.zip › S0007125021000969sup002.docx]

**Supplementary material**

**Supplemental 1.** Deviations from protocol

1. We used a quality assessment tool that was developed to assess ethnic density studies specifically (see Supplemental 6) and GRADE assessments (Supplemental 7) rather than the assessment specified in the protocol.
2. In the protocol it was not specified that a three-level meta-analysis would be used. The justification for using this method rather than a method of meta-analysis that does not account for the hierarchical structure of data is outlined in the main paper.
3. Methods used to categorise minority group samples were not detailed in the protocol. We also did not specify how we would decide which studies to include if there were overlapping datasets. Justifications for both are outlined in the main paper.
4. Our eligibility criteria were refined to only include within-groups ethnic density studies *i.e.,* those that compared risk within the ethnic minority group between ethnic density exposures, as opposed to between-groups studies which compare risk between the ethnic minority and majority at different ethnic density exposures. It would not have been appropriate to combine within- and between-group studies in the meta-analysis. We opted to only include the former given evidence of social drift in majority groups into high ethnic density areas (e.g., Termorshuizen et al., 2014) which may result in artefactually stronger between-group effect sizes. To derive a more reliable estimate, we also decided to only include studies that used multilevel modelling to account for non-independence of data and studies that adjusted for individual- and area-level confounds (minimally age, sex, and area-level deprivation).
5. The protocol did not specify that effect sizes would be standardised. This decision was made after observing that included studies measured exposure differently which presented challenges for the meta-analysis. Rather than excluding studies based on how they quantified group density, we chose to rescale each effect size to estimate the response in risk to a ten percentage-point decrease in group density. Ten percentage-point was used because this was a common method used by included studies. Strengths and limitations of this approach have been outlined in the main paper.

**Supplemental 2.** Full list of search terms

1. **Population**

Psychosis*

Psychotic*

Schizophrenia

Schizoaffective*

Bipolar*

“Manic depress*”

“Severe mental illness”

“Mental distress”

Hallucinat*

Delusion*

Paranoi*

1. **Ethnic density terms**

Minorit*

“Ethnic density”

“Ethnic enclave”

“Ethnic composition”

“Group density”

1. **Outcome measures**

Incidence

Prevalence

ICD*

DSM*

PANSS

CAARMS

SIPS

PSQ

Symptom*

“Psycho* proneness”

“Psycho* experiences”

“Psycho* syndrome”

“Psycho* disorder”

“Psycho* risk”

“Ultra-high risk”

“At-risk mental state”

1. **Geographical terms**

Neighbo*

Residential

County

Local*

Area

Zone

District

Ecological

Geograph*

Community

Municipal

Spatial

State

Tract

“Electoral ward”

“Output area”

“Dissemination area”

**Combine each term in A, B, C, D with OR**

**Then A AND B AND C AND D AND E**

**Supplemental 3.** PsycINFO search

| Set# | Searched for | Databases | Results |
| --- | --- | --- | --- |
| S1 | MAINSUBJECT.EXACT.EXPLODE("Psychosis") | PsycINFO | 111926 |
| S2 | Psychotic* | PsycINFO | 99406 |
| S3 | Schizophrenia* | PsycINFO | 141692 |
| S4 | Schizoaffective* | PsycINFO | 6880 |
| S5 | Bipolar* | PsycINFO | 47111 |
| S6 | “Manic depress*” | PsycINFO | 5157 |
| S7 | “Severe mental illness” | PsycINFO | 4871 |
| S8 | “Mental distress” | PsycINFO | 1548 |
| S9 | Hallucinat* | PsycINFO | 16724 |
| S10 | Delusion* | PsycINFO | 16174 |
| S11 | Paranoi* | PsycINFO | 16040 |
| S12 | S1 OR S2 OR S3 OR S4 OR S4 OR S6 OR S7 OR S8 OR S9 OR S10 OR S11 | PsycINFO  These databases are searched for part of your query. | 203429 |
| S13 | MAINSUBJECT.EXACT.EXPLODE("Minority Groups") | PsycINFO | 14945 |
| S14 | Minorit* | PsycINFO | 57035 |
| S15 | "Ethnic density" | PsycINFO | 151 |
| S16 | "Ethnic enclave" | PsycINFO | 93 |
| S17 | "Ethnic composition" | PsycINFO | 644 |
| S18 | "Group density" | PsycINFO | 61 |
| S19 | S14 OR S15 OR S16 OR S17 OR S18 | PsycINFO  These databases are searched for part of your query. | 57714 |
| S20 | Incidence | PsycINFO | 58024 |
| S21 | Prevalence | PsycINFO | 119359 |
| S22 | ICD* | PsycINFO | 9800 |
| S23 | DSM* | PsycINFO | 81940 |
| S24 | PANSS | PsycINFO | 3461 |
| S25 | CAARMS | PsycINFO | 91 |
| S26 | SIPS | PsycINFO | 1146 |
| S27 | PSQ | PsycINFO | 322 |
| S28 | Symptom* | PsycINFO | 341715 |
| S29 | "Psycho* proneness" | PsycINFO | 393 |
| S30 | “Psycho* experiences” | PsycINFO | 914 |
| S31 | “Psycho* syndrome” | PsycINFO | 278 |
| S32 | “Psycho* disorder” | PsycINFO | 3228 |
| S33 | “Psycho* risk” | PsycINFO | 585 |
| S34 | “Ultra-high risk” | PsycINFO | 861 |
| S35 | “At-risk mental state” | PsycINFO | 467 |
| S36 | S20 OR S21 OR S22 OR S23 OR S24 OR S25 OR S26 OR S27 OR S28 OR S29 OR S30 OR S31 OR S32 OR S33 OR S34 OR S35 | PsycINFO  These databases are searched for part of your query. | 525155 |
| S37 | Neighbo* | PsycINFO | 26715 |
| S38 | Residential | PsycINFO | 36892 |
| S39 | County | PsycINFO | 32611 |
| S40 | Local* | PsycINFO | 118264 |
| S41 | Area | PsycINFO | 340290 |
| S42 | Zone | PsycINFO | 15557 |
| S43 | District | PsycINFO | 40986 |
| S44 | Ecological | PsycINFO | 32296 |
| S45 | Geograph* | PsycINFO | 30787 |
| S46 | Community | PsycINFO | 380131 |
| S47 | Municipal | PsycINFO | 5491 |
| S48 | Spatial | PsycINFO | 98692 |
| S49 | State | PsycINFO | 933384 |
| S50 | Tract | PsycINFO | 18706 |
| S51 | “Electoral ward” | PsycINFO | 18 |
| S52 | “Output area” | PsycINFO | 35 |
| S53 | “Dissemination area” | PsycINFO | 4 |
| S54 | S37 OR S38 OR S39 OR S40 OR S41 OR S42 OR S43 OR S44 OR S45 OR S46 OR S47 OR S48 OR S49 OR S50 OR S51 OR S52 OR S53 | PsycINFO  These databases are searched for part of your query. | 1679313 |
| S55 | S12 AND S19 AND S36 AND S54 | PsycINFO  These databases are searched for part of your query. | 456 |

**Supplemental 4.** Screening for non-English papers (n=14)

| Author | Paper | Language | Eligible for narrative review or meta-analysis? |
| --- | --- | --- | --- |
| Adriaanse et al., (2018) | Psychotische ervaringen bij jeugdigen met een migratieachtergrond: Prevalentie, impact en culturele context *(English translation: Prevalence, impact and cultural context of psychotic experiences among ethnic minority youth)* | Dutch | Exclude – no group density analyses |
| Binbay et al., (2016) | Yeni Bir Sosyal Ortama Uyum Sürecinde Psikotik Yaşantılar *(English translation: Psychotic Experiences in the Adaptation Process to a New Social Environment)* | Turkish | Exclude – no group density analyses |
| Chapireau (2005) | Les nouveaux longs séjours en établissements de soins spécialisés en psychiatrie : résultats d’une enquête nationale sur un échantillon représentatif (1998-2000) *(English translation: Old and new long stay patients in French psychiatric institutions : results from a national random survey with two-year follow-up (1998-2000))* | French | Exclude – outcomes not specific to psychosis, no group density analyses |
| Egea et al., (2004) | Trastorno esquizofreniforme. Estudio prospective de 5 años de seguimiento *(English translation: Schizophreniform disorder. A five year prospective study)* | Spanish | Exclude – no group density analyses |
| Faerden, Waal, & Rønnow (1995) | Langsiktige psykiatriske pasienter i en sektor av Oslo *(English translation: Long-term psychiatric patients in a sector of Oslo)* | Norwegian | Exclude – outcomes not specific to psychosis, no group density analyses |
| Hódi (1989) | A pszichózisok és öngyilkosságok területi és etnikai megoszlása a Vajdaságban *(English translation: The regional and ethnic distribution of psychoses and suicides in Voivodina Province, Yugoslavia).* | Hungarian | Exclude – no group density analyses |
| Wenxing et al., (2015) | 云南省西盟佤族自治县精神障碍现况调查 *(English translation: A cross-sectional study of mental disorders in Ximeng Wa Autonomous County of Yunnan Province)* | Chinese | Exclude – no group density analyses |
| Melle et al., (2016) | Verbesserung der Ergebnisse: Einflussfaktoren auf das Hilfesuchverhalten von Zuwanderern und ethnischen Minderheiten mit psychotischen Ersterkrankungen *(English translation: Improving outcomes: Factors influencing help-seeking behaviors in immigrants and ethnic minorities with first-episode psychosis)* | German | Exclude – no primary data (confirmed by author) |
| Meurice et al., (2013) | Peut-on prédire, dès l’enfance, les risques de développer la schizophrénie à l’âge adulte ? Une étude rétrospective centrée sur l’hypersensibilité prémorbide; premiers résultats *(English translation: Is it possible to predict, as early as childhood, the risk of developing schizophrenia in adulthood? A retrospective study focused on premorbid hypersensitivity; first results)* | French | Exclude – no group density analyses |
| Mena et al., (2002) | Estudio descriptivo de trastornos mentales en minorías étnicas residentes en un área urbana de Barcelona *(English translation: Descriptive study of mental disorders in ethnic minorities residing in an urban area of Barcelona)* | Spanish | Exclude – no group density analyses |
| Picarda & Ineichen (1995) | La santé mentale des minorités ethniques au Royaume-Uni *(English translation: The mental health of ethnic minorities in the United Kingdom)* | French | Exclude – review paper, no primary data, no new papers found in reference list |
| Plancke & Amariei (2017) | Hospitalisations psychiatriques de longue durée *(English translation: Long-term psychiatric hospitalizations)* | French | Exclude – no group density analyses, outcomes not specific to psychosis |
| van der Stoep (2016) | Culturele diversiteit in de forensische  psychiatrie; een exploratief onderzoek  in Forensisch Psychiatrisch Centrum  de Oostvaarderskliniek *(English translation: Cultural diversity in the forensics*  *psychiatry; an exploratory study*  *in Forensic Psychiatric Center*  *the Oostvaarders clinic)* | Dutch | Exclude – no group density analyses |
| Vilain et al., (2013) | Les facteurs de risque environnementaux de la schizophrénie *(English translation: Environmental risk factors for schizophrenia: A review)* | French | Exclude – review paper, no primary data, no new papers found in reference list |
|  |  |  |  |

**Supplemental 5.** Example of calculations for rescaling effect sizes

“African” group from Schofield, Thygesen, Das-Munshi et al., (2017)

To rescale effect sizes and their corresponding 95% confidence intervals, we extracted the ethnic density percentage for each category, *e.g.,* for the African group (Schofield, Thygesen, Das-Munshi et al., 2017), this was 3.7-18.5% (REF/highest quintile), 1.7-3.7% (4th quintile), 0.9-1.7% (3rd quintile), 0.4-0.9% (2nd quintile), and <0.4% (1st/lowest quintile). We took a mid-point or given estimate for each category (in this case: 11.1, 2.7, 1.3, 0.65, 0.3) and subtracted each of these values from the reference category. Taking the effect size for the 4th quintile as an example, we subtracted 2.7 (midpoint for 4th quintile) from 11.1 (midpoint for 1st quintile/REF category) which gave 8.4. We then wanted to rescale the raw effect size to reflect the response in risk to a 10% reduction in ethnic density so we used the following formula: exp[Log[1.20]*(10/8.4)] which gives a rescaled effect size of 1.24. We used the same formulae to calculate the confidence intervals (0.77-2.01). These values were then converted to their natural logarithmic form, from which log standard errors and sampling variances were computed. We followed these steps to rescale all 75 effect sizes unless of course, the papers already reported effect sizes associated with a ten-percentage point decrease in ethnic density *e.g.,* Das-Munshi, Bécares, Boydell et al., (2012).

**Supplemental 6.** Quality assessment checklist for ethnic density studies taken from Bécares, Dewey, & Das-Munshi (2018)

| **Quality criteria** | **Score** |
| --- | --- |
| **Type of study** |  |
| Cross-sectional, case-control | 1 |
| Cohort | 2 |
| **Exposure** |  |
| Explicitly defines ethnic density exposure | 1 |
| **Ethnicity** |  |
| Self-ascribed ethnicity | 1 |
| **Language of interview** |  |
| Language of interview in own language if English not preferred language | 1 |
| **Outcome** |  |
| Assessed with structured instrument | 1 |
| Validated instrument used to assess outcome | 1 |
| Reliable instrument used to assess outcome | 1 |
| Instrument validated in racial/ ethnic group | 1 |
| Outcome assessment blind to exposure status | 1 |
| **Sample size and power calculation** |  |
| n>500 | 1 |
| **Response rates** |  |
| For case-control/ cross-sectional studies – Response rates |  |
| >60% | 1 |
| For cohort studies – Rate of attrition compared to baseline |  |
| >60% | 1 |
| Response rates similar across racial/ ethnic groups or weighted to allow for differential non-response | 1 |
| **Methods** |  |
| Estimate available adjusted for area-level deprivation  Adjusts with one variable for area-level deprivation  Adjusts with composite measure of area-level deprivation | 1  2 |
| Appropriate statistical methods used (e.g. multi-level modelling or robust standard errors to account for clustered data) | 1 |
| Cohort studies - Assesses dose-response | 1 |

**Supplemental 7.** GRADE assessments

GRADE assessment table for psychosis outcomes:

| **Group** | **No. of studies** | **Study design** | **Risk of bias** | **Inconsistency** | **Indirectness** | **Imprecision** | **Other considerations** | **Pooled ES (95% CI) for subgroup** ^[[1]](#footnote-1)^ | **Certainty** |
| --- | --- | --- | --- | --- | --- | --- | --- | --- | --- |
| **Non-affective psychosis** | 5 | 2 observational  3 longitudinal | Not serious | Not serious | Not serious | Not serious | Dose response gradient | **OR 1.14** (1.04 to 1.25) | ⨁⨁⨁◯  MODERATE |
| **Subclinical psychosis** | 3 | 3 observational | Not serious | Not serious | Not serious | Not serious | Dose response gradient | **OR 1.12** (0.96 to 1.31) | ⨁⨁⨁◯  MODERATE |
| **Antipsychotic prescriptions** | 1 | 1 observational | Not serious | Not serious | Serious ^[[2]](#footnote-2)^ | Not serious | Dose response gradient | **OR 1.04** (0.95 to 1.17) | ⨁⨁◯◯  LOW |
| **Any psychosis** | 1 | 1 observational | Not serious | Not serious | Not serious | Serious ^[[3]](#footnote-3)^ | Strong association  Dose response gradient | **OR 1.90** (1.43 to 2.53) | ⨁⨁⨁◯  MODERATE |
| **Affective psychosis** | 2 | 1 observational  1 longitudinal | Not serious | Not serious | Not serious | Not serious | Dose response gradient | **OR 1.16** (1.04 to 1.28) | ⨁⨁⨁◯  MODERATE |
| **Other psychoses** | 1 | Longitudinal | Not serious | Not serious | Not serious | Not serious | Dose response gradient | **OR 1.07** (1.00 to 1.16) | ⨁⨁⨁◯  MODERATE |

GRADE assessment table for crude minority subgroups:

| **Group** | **No. of studies** | **Study design** | **Risk of bias** | **Inconsistency** | **Indirectness** | **Imprecision** | **Other considerations** | **Pooled ES (95% CI) for subgroup** ^[[4]](#footnote-4)^ | **Certainty** |
| --- | --- | --- | --- | --- | --- | --- | --- | --- | --- |
| **Black** | 7 | 5 observational  2 longitudinal | Not serious | Not serious | Serious ^[[5]](#footnote-5)^ | Not serious | Strong association, dose response gradient | **OR 1.62** (1.17 to 2.24) | ⨁⨁⨁◯ MODERATE |
| **Asian** | 4 | 2 observational  2 longitudinal | Not serious | Not serious | Not serious | Not serious | Dose response gradient | **OR 1.16** (0.93 to 1.45) | ⨁⨁⨁◯ MODERATE |
| **White other** | 3 | observational studies | Not serious | Not serious | Not serious | Serious ^[[6]](#footnote-6)^ | Dose response gradient | **OR 1.24** (0.77 to 1.97) | ⨁⨁◯◯  LOW |
| **Other ethnic group** | 3 | 1 observational  1 longitudinal | Not serious | Not serious | Serious ^[[7]](#footnote-7)^ | Not serious | Dose response gradient | **OR 1.07** (0.97 to 1.17) | ⨁⨁◯◯  LOW |
| **Combined migrant group** | 2 | 1 observational  1 longitudinal | Serious ^[[8]](#footnote-8)^ | Not serious | Not serious | Not serious | Dose response gradient | **OR 1.08** (1.00 to 1.16) | ⨁⨁◯◯  LOW |
| **Combined ethnic minority group** | 1 | observational study | Not serious | Not serious | Not serious | Serious ^[[9]](#footnote-9)^ |  | **OR 1.13** (0.63 to 2.03) | ⨁⨁◯◯  LOW |
| **Other social characteristic** | 2 | 1 observational  1 longitudinal | Not serious | Not serious | Not serious | Not serious | Dose response gradient | **OR 1.02** (0.86 to 1.20) | ⨁⨁⨁◯ MODERATE |

**Supplemental 8.** Output showing full model and model fit after each of the levels were removed

Full model:


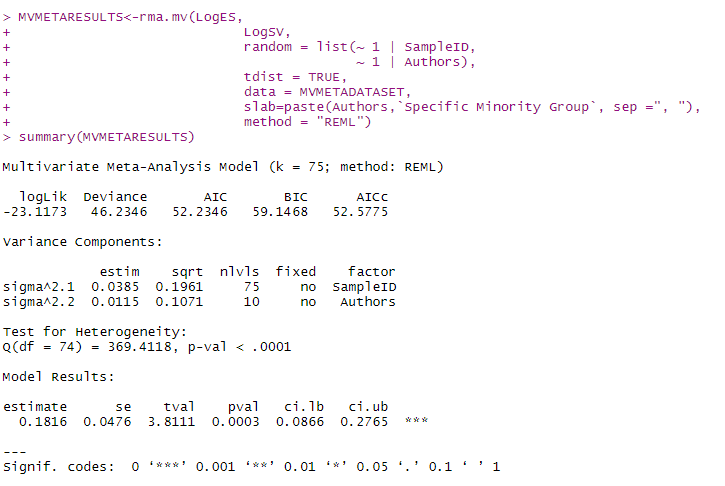


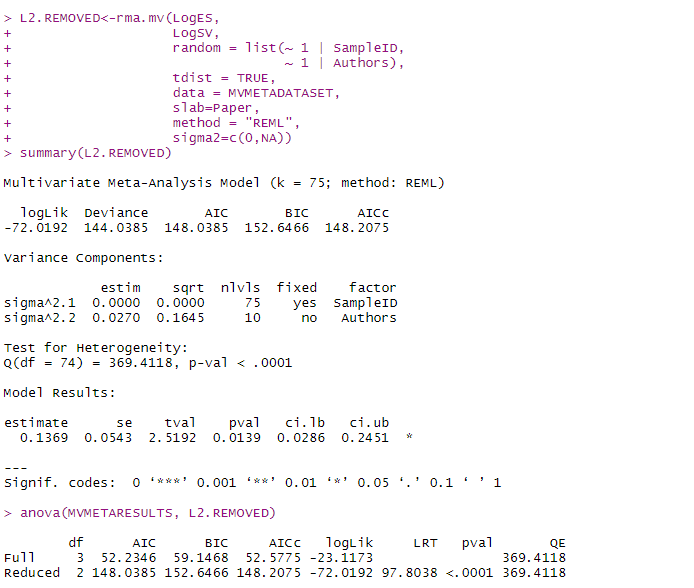
Level two removed:

Level 3 removed:

Level 3 removed:


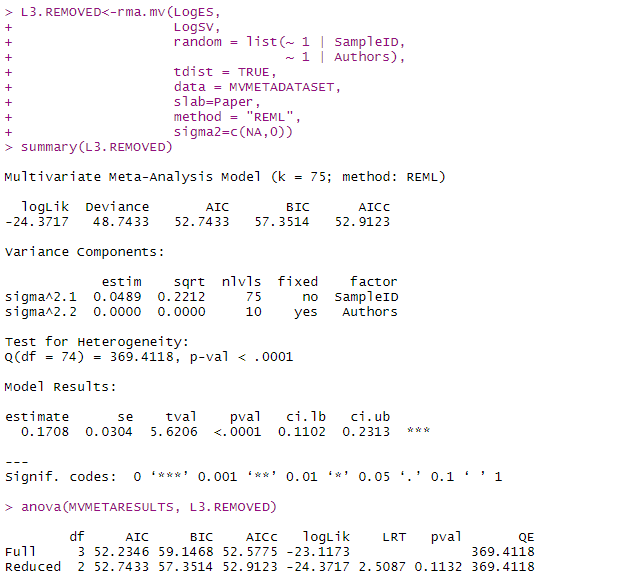
Level 3 removed:

**Supplemental 9.** Crude and specific minority moderator tests for ethnic minorities and migrants only


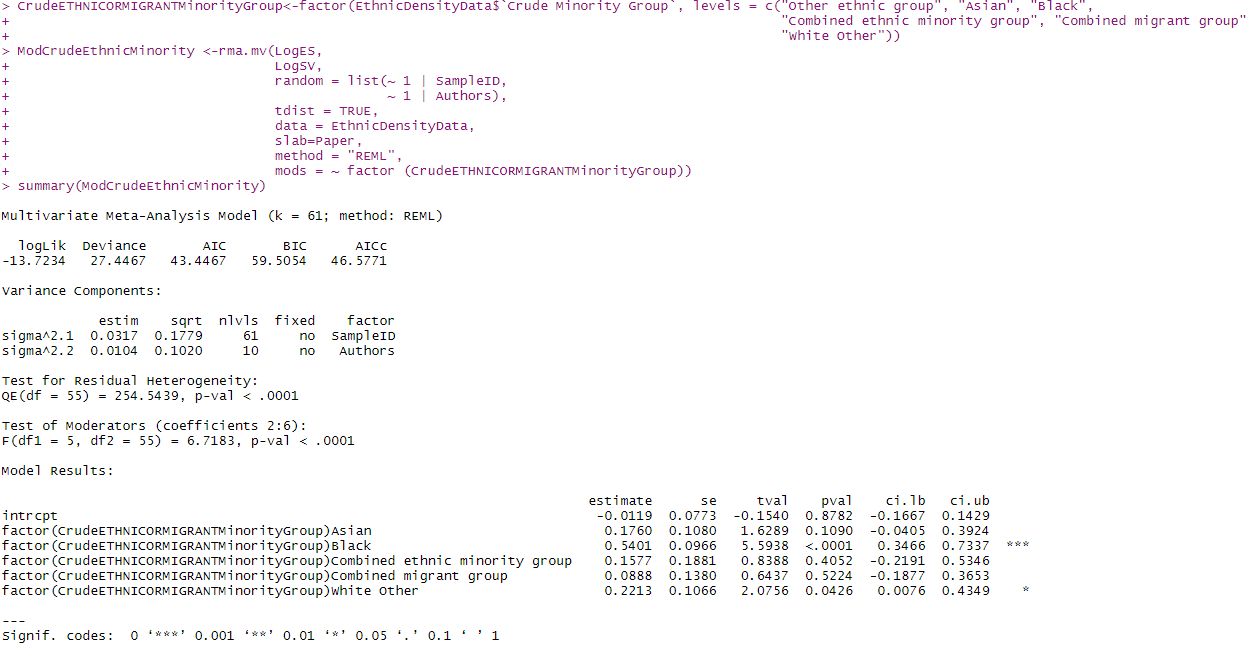
Crude:

Specific:


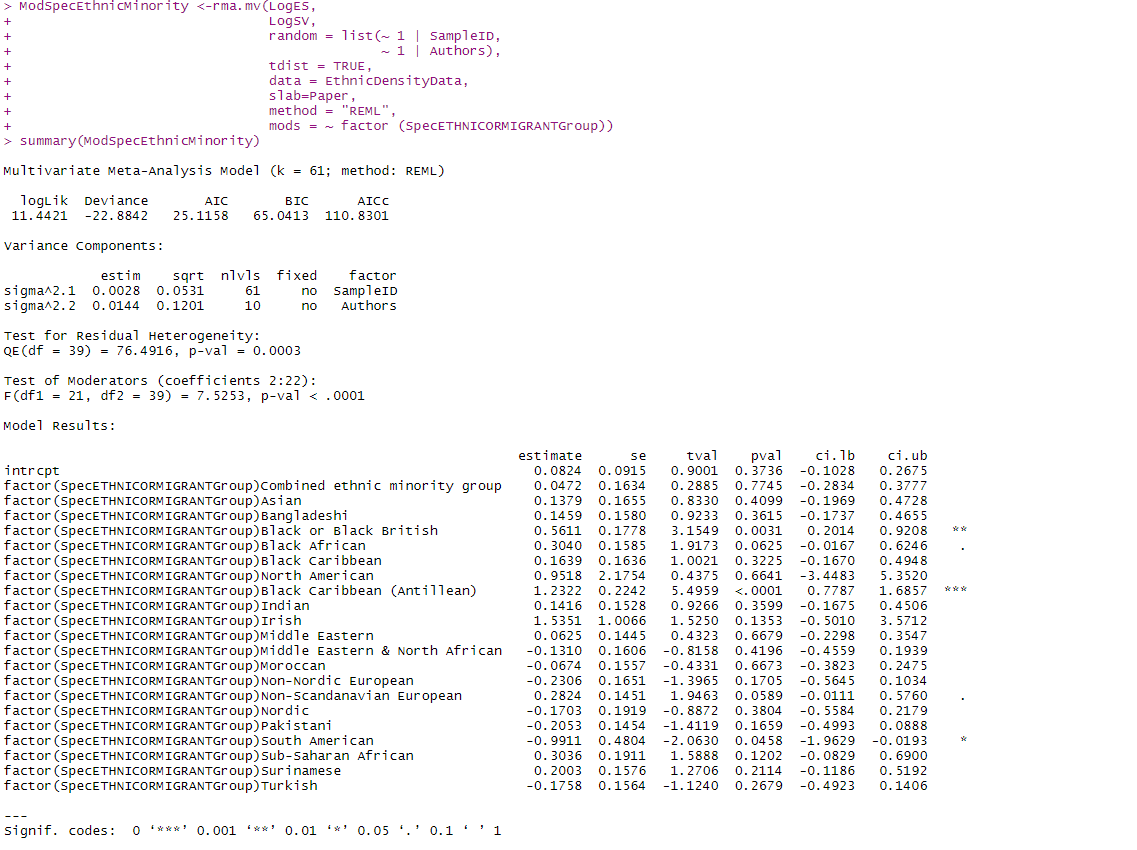


**Supplemental 10.** Moderator test results for specific minority groups (all samples)


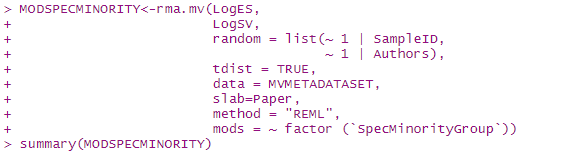


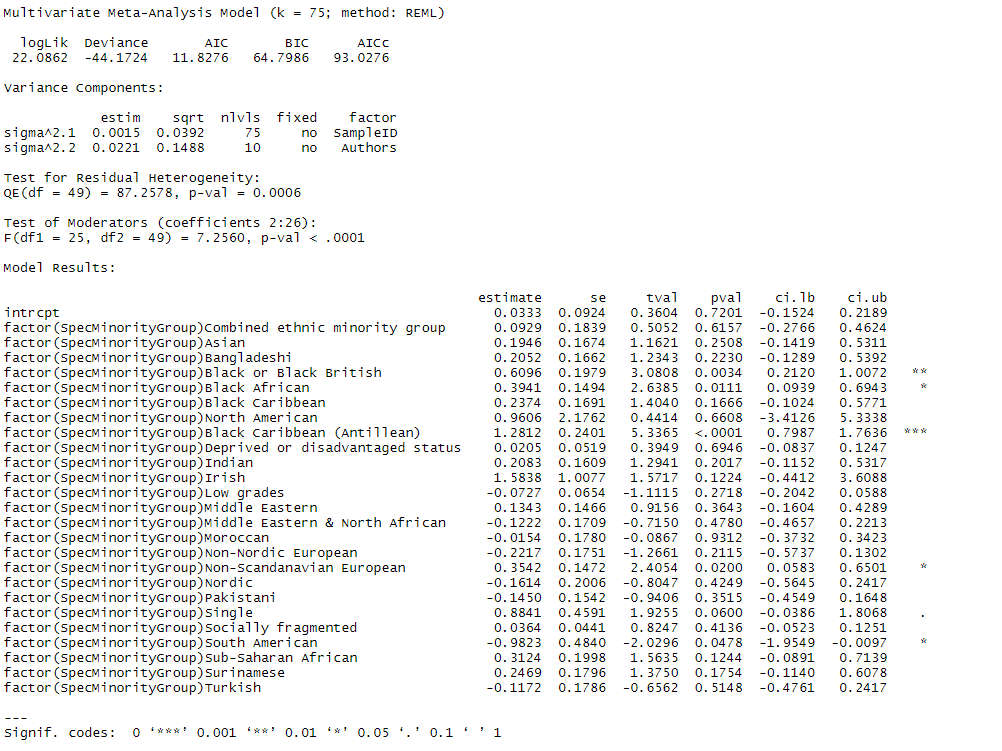


**Supplemental 11.** Leave-one-out analysis for individual samples (*n*=75)

| **Sample ID** | **Paper** | **Estimates** | **LBs** | **UBs** | **p.vals** |
| --- | --- | --- | --- | --- | --- |
| 1 | Bécares et al., (2009) - Black Caribbean - Subclinical | 1.197424274 | 1.084689182 | 1.321876 | 0.00052 |
| 2 | Bécares et al., (2009) - Indian - Subclinical | 1.200092336 | 1.089610839 | 1.321776 | 0.000335 |
| 3 | Bécares et al., (2009) - Pakistani - Subclinical | 1.211046497 | 1.106794029 | 1.325119 | 6.47E-05 |
| 4 | Bécares et al., (2009) - Bangladeshi - Subclinical | 1.196901168 | 1.083901406 | 1.321681 | 0.000554 |
| 5 | Das-Munshi et al., (2012) - Irish - Subclinical | 1.196708706 | 1.08872372 | 1.315404 | 0.000313 |
| 6 | Das-Munshi et al., (2012) - Black Caribbean - Subclinical | 1.202720102 | 1.091641903 | 1.325101 | 0.000301 |
| 7 | Das-Munshi et al., (2012) - Bangladeshi - Subclinical | 1.198875711 | 1.088691361 | 1.320212 | 0.000352 |
| 8 | Das-Munshi et al., (2012) - Indian - Subclinical | 1.19610088 | 1.086843051 | 1.316342 | 0.000381 |
| 9 | Das-Munshi et al., (2012) - Pakistani -Subclinical | 1.201666787 | 1.09033815 | 1.324363 | 0.000333 |
| 10 | Dykxhoorn, Lewis, Hollander, Kirkbride & Dalman (2020) - Nordic | 1.203677629 | 1.093592878 | 1.324844 | 0.000249 |
| 11 | Dykxhoorn, Lewis, Hollander, Kirkbride & Dalman (2020) - Non-Nordic European | 1.206339827 | 1.095070549 | 1.328915 | 0.00024 |
| 12 | Dykxhoorn, Lewis, Hollander, Kirkbride & Dalman (2020) - Asian | 1.189852361 | 1.07821423 | 1.313049 | 0.000756 |
| 13 | Dykxhoorn, Lewis, Hollander, Kirkbride & Dalman (2020) - Middle Eastern & North African | 1.203726996 | 1.093298772 | 1.325309 | 0.000259 |
| 14 | Dykxhoorn, Lewis, Hollander, Kirkbride & Dalman (2020) - Sub-Saharan African | 1.188914515 | 1.076322354 | 1.313285 | 0.000888 |
| 15 | Dykxhoorn, Lewis, Hollander, Kirkbride & Dalman (2020) - North American | 1.198842474 | 1.090203597 | 1.318307 | 0.000292 |
| 16 | Dykxhoorn, Lewis, Hollander, Kirkbride & Dalman (2020) - South American | 1.203854218 | 1.0944829 | 1.324155 | 0.000225 |
| 17 | Menezes et al., (2011) - Migrant status - SZ | 1.204734747 | 1.089008711 | 1.332759 | 0.00045 |
| 18 | Richardson et al., (2018) - Overall minority group - NAP | 1.204233366 | 1.091097925 | 1.3291 | 0.000347 |
| 19 | Richardson et al., (2018) - Overall minority group -AP | 1.198759509 | 1.08687213 | 1.322165 | 0.000433 |
| 20 | Schofield et al., (2011) - Black - High ethnic density - Any psychosis | 1.188286563 | 1.095643057 | 1.288764 | 6.56E-05 |
| 21 | Schofield et al., (2011) - Black - Mid ethnic density - Any psychosis | 1.185638519 | 1.095777841 | 1.282868 | 5.1E-05 |
| 22 | Schofield et al., (2011) - Black - Low ethnic density - Any psychosis | 1.183345833 | 1.095826208 | 1.277855 | 4.1E-05 |
| 23 | Schofield et al., (2011) - Black - Lowest ethnic density - Any psychosis | 1.191329876 | 1.094422673 | 1.296818 | 0.000101 |
| 24 | Schofield et al., (2016) - Black African - Subclinical | 1.200582784 | 1.089868981 | 1.322543 | 0.000334 |
| 25 | Schofield et al., (2016) - Black Caribbean - Subclinical | 1.195122676 | 1.085041015 | 1.316373 | 0.000449 |
| 26 | Schofield et al., (2016) - Disadvantaged social class - Subclinical | 1.213949632 | 1.097647762 | 1.342574 | 0.000263 |
| 27 | Schofield et al., (2016) - Single household status - Subclinical | 1.194147187 | 1.083685884 | 1.315868 | 0.000501 |
| 28 | Schofield et al., (2017) - African - High ethnic density - NAP | 1.199594665 | 1.090863685 | 1.319163 | 0.00028 |
| 29 | Schofield et al., (2017) - African - Mid ethnic density - NAP | 1.200557618 | 1.091128951 | 1.320961 | 0.000286 |
| 30 | Schofield et al., (2017) - African - Low ethnic density - NAP | 1.192942945 | 1.086721759 | 1.309547 | 0.000328 |
| 31 | Schofield et al., (2017) - African - Lowest ethnic density - NAP | 1.19570159 | 1.088820934 | 1.313074 | 0.000293 |
| 32 | Schofield et al., (2017) - Non-Scandanavian European - High ethnic density - NAP | 1.197605453 | 1.090075444 | 1.315743 | 0.000278 |
| 33 | Schofield et al., (2017) - Non-Scandanavian European - Mid ethnic density - NAP | 1.199470709 | 1.090998445 | 1.318728 | 0.000274 |
| 34 | Schofield et al., (2017) - Non-Scandanavian European - Low ethnic density - NAP | 1.198112541 | 1.090367756 | 1.316504 | 0.000275 |
| 35 | Schofield et al., (2017) - Non-Scandanavian European - Lowest ethnic density - NAP | 1.195127947 | 1.088462621 | 1.312246 | 0.000297 |
| 36 | Schofield et al., (2017) - Asian - High ethnic density - NAP | 1.201147448 | 1.091152846 | 1.32223 | 0.000294 |
| 37 | Schofield et al., (2017) - Asian - Mid ethnic density - NAP | 1.201907692 | 1.091070536 | 1.324004 | 0.000309 |
| 38 | Schofield et al., (2017) - Asian - Low ethnic density - NAP | 1.201214287 | 1.091164715 | 1.322363 | 0.000295 |
| 39 | Schofield et al., (2017) - Asian - Lowest ethnic density - NAP | 1.196761082 | 1.089487527 | 1.314597 | 0.000286 |
| 40 | Schofield et al., (2017) - Middle Eastern - High ethnic density - NAP | 1.201745699 | 1.091505585 | 1.32312 | 0.000291 |
| 41 | Schofield et al., (2017) - Middle Eastern - Mid ethnic density - NAP | 1.202810381 | 1.091159592 | 1.325886 | 0.00032 |
| 42 | Schofield et al., (2017) - Middle Eastern- Low ethnic density - NAP | 1.201810558 | 1.091528616 | 1.323235 | 0.000291 |
| 43 | Schofield et al., (2017) - Middle Eastern - Lowest ethnic density - NAP | 1.199943168 | 1.091180311 | 1.319547 | 0.000275 |
| 44 | Termorshuizen et al., (2018) - Turkish - High ethnic density - Antipsychotic prescriptions | 1.203087228 | 1.093213349 | 1.324004 | 0.000253 |
| 45 | Termorshuizen et al., (2018) - Turkish - Mid ethnic density - Antipsychotic prescriptions | 1.203610931 | 1.093343905 | 1.324999 | 0.000256 |
| 46 | Termorshuizen et al., (2018) - Turkish - Low ethnic density - Antipsychotic prescriptions | 1.203508814 | 1.093834351 | 1.32418 | 0.00024 |
| 47 | Termorshuizen et al., (2018) - Turkish - Lowest ethnic density - Antipsychotic prescriptions | 1.203217357 | 1.09374995 | 1.323641 | 0.000238 |
| 48 | Termorshuizen et al., (2018) - Moroccan - High ethnic density - Antipsychotic prescriptions | 1.201005684 | 1.092201303 | 1.320649 | 0.000256 |
| 49 | Termorshuizen et al., (2018) - Moroccan - Mid ethnic density - Antipsychotic prescriptions | 1.203339184 | 1.093777347 | 1.323876 | 0.000239 |
| 50 | Termorshuizen et al., (2018) - Moroccan - Low ethnic density - Antipsychotic prescriptions | 1.202543675 | 1.093361852 | 1.322628 | 0.000241 |
| 51 | Termorshuizen et al., (2018) - Moroccan - Lowest ethnic density - Antipsychotic prescriptions | 1.202397349 | 1.093260909 | 1.322428 | 0.000242 |
| 52 | Termorshuizen et al., (2018) - Surinamese - High ethnic density - Antipsychotic prescriptions | 1.192375025 | 1.082514616 | 1.313385 | 0.000527 |
| 53 | Termorshuizen et al., (2018) - Surinamese - Mid ethnic density - Antipsychotic prescriptions | 1.197478394 | 1.08860035 | 1.317246 | 0.000331 |
| 54 | Termorshuizen et al., (2018) - Surinamese - Low ethnic density - Antipsychotic prescriptions | 1.200301009 | 1.091504171 | 1.319942 | 0.000269 |
| 55 | Termorshuizen et al., (2018) - Surinamese - Lowest ethnic density - Antipsychotic prescriptions | 1.1963054 | 1.087192119 | 1.31637 | 0.000369 |
| 56 | Termorshuizen et al., (2018) - Antillean - High ethnic density - Antipsychotic prescriptions | 1.198656216 | 1.090035599 | 1.318101 | 0.000296 |
| 57 | Termorshuizen et al., (2018) - Antillean - Mid ethnic density - Antipsychotic prescriptions | 1.194727694 | 1.085528269 | 1.314912 | 0.000416 |
| 58 | Termorshuizen et al., (2018) - Antillean - Low ethnic density - Antipsychotic prescriptions | 1.189902843 | 1.079306545 | 1.311832 | 0.000674 |
| 59 | Termorshuizen et al., (2018) - Antillean - Lowest ethnic density - Antipsychotic prescriptions | 1.184120226 | 1.071832397 | 1.308172 | 0.001164 |
| 60 | Zammit et al., (2010) - Migrant status - NAP | 1.200938676 | 1.093541232 | 1.318884 | 0.000215 |
| 61 | Zammit et al., (2010) - Migrant status - AP | 1.199727832 | 1.09069797 | 1.319657 | 0.000288 |
| 62 | Zammit et al., (2010) - Migrant status - SZ | 1.200389611 | 1.092191228 | 1.319307 | 0.000248 |
| 63 | Zammit et al., (2010) - Migrant status - Other | 1.200428084 | 1.092242738 | 1.319329 | 0.000247 |
| 64 | Zammit et al., (2010) - Socially fragmented - NAP | 1.200616293 | 1.092737024 | 1.319146 | 0.000234 |
| 65 | Zammit et al., (2010) - Socially fragmented - AP | 1.198384969 | 1.088089957 | 1.31986 | 0.000369 |
| 66 | Zammit et al., (2010) - Socially fragmented - SZ | 1.199842192 | 1.090996199 | 1.319547 | 0.00028 |
| 67 | Zammit et al., (2010) - Socially fragmented - Other | 1.200024152 | 1.091332208 | 1.319541 | 0.000271 |
| 68 | Zammit et al., (2010) - Deprived - NAP | 1.200212626 | 1.091840045 | 1.319342 | 0.000257 |
| 69 | Zammit et al., (2010) - Deprived - AP | 1.198502777 | 1.088405795 | 1.319737 | 0.000357 |
| 70 | Zammit et al., (2010) - Deprived - SZ | 1.200726735 | 1.093304114 | 1.318704 | 0.000219 |
| 71 | Zammit et al., (2010) - Deprived - Other | 1.19998113 | 1.091294803 | 1.319492 | 0.000271 |
| 72 | Zammit et al., (2010) - Low grades - NAP | 1.20117337 | 1.094621132 | 1.318098 | 0.000189 |
| 73 | Zammit et al., (2010) - Low grades - AP | 1.200628663 | 1.093225531 | 1.318584 | 0.00022 |
| 74 | Zammit et al., (2010) - Low grades - SZ | 1.200057175 | 1.091826955 | 1.319016 | 0.000255 |
| 75 | Zammit et al., (2010) - Low grades - Other | 1.200631333 | 1.092936704 | 1.318938 | 0.000229 |

1. Estimates not from original papers, these represent the recalculated ESs & CIs for subgroup i.e., each estimate has been standardised to reflect 10% decrease in group density [↑](#footnote-ref-1)
2. Outcome used in one large scale study (Termorshuizen et al., 2018) - antipsychotic prescriptions [↑](#footnote-ref-2)
3. Large ES, Wide CIs and only one study in subgroup [↑](#footnote-ref-3)
4. Estimates not from original papers, these represent the recalculated ESs & CIs for subgroup i.e., each estimate has been standardised to reflect 10% decrease in group density [↑](#footnote-ref-4)
5. Outcome used in one large scale study (Termorshuizen et al., 2018) - antipsychotic prescriptions [↑](#footnote-ref-5)
6. Wide CIs for subgroup’s pooled estimate [↑](#footnote-ref-6)
7. Outcome used in one large scale study (Termorshuizen et al., 2018) - antipsychotic prescriptions [↑](#footnote-ref-7)
8. Concerns regarding the reliability and validity of the psychosis measure in one study (Menezes, Georgiades, & Boyle, 2011) - self-reported lifetime prevalence of diagnosed Schizophrenia or other psychosis [↑](#footnote-ref-8)
9. Wide CIs for subgroup’s pooled estimate and only one study [↑](#footnote-ref-9)
